# Reproducible phenotype alteration due to prolonged cooling of the pupae of *Polyommatus icarus* butterflies

**DOI:** 10.1371/journal.pone.0225388

**Published:** 2019-11-25

**Authors:** Gábor Piszter, Krisztián Kertész, Zsolt Endre Horváth, Zsolt Bálint, László Péter Biró

**Affiliations:** 1 Institute of Technical Physics and Materials Science, Centre for Energy Research, Budapest, Hungary; 2 Hungarian Natural History Museum, Budapest, Hungary; USDA Agricultural Research Service, UNITED STATES

## Abstract

The phenotypic changes induced by prolonged cooling (2–12 weeks at 5 °C in the dark) of freshly formed *Polyommatus icarus* pupae were investigated. Cooling halted the imaginal development of pupae collected shortly after transformation from the larval stage. After cooling, the pupae were allowed to continue their developmental cycle. The wings of the eclosed specimens were investigated by optical microscopy, scanning and cross-sectional transmission electron microscopy, UV-VIS spectroscopy and microspectroscopy. The eclosed adults presented phenotypic alterations that reproduced results that we published previously for smaller groups of individuals remarkably well; these changes included i) a linear increase in the magnitude of quantified deviation from normal ventral wing patterns with increasing cooling time; ii) slight alteration of the blue coloration of males; and iii) an increasing number of blue scales on the dorsal wing surface of females with increasing cooling time. Several independent factors, including disordering of regular scale rows in males, the number of blue scales in females, eclosion probability and the probability of defect-free eclosion, showed that the cooling time can be divided into three periods: 0–4 weeks, 4–8 weeks, and 8–12 weeks, each of which is characterized by specific changes. The shift from brown female scales to first blue scales with a female-specific shape and then to blue scales with a male-specific shape with longer cooling times suggests slow decomposition of a substance governing scale formation.

## Introduction

The field of integrative biology, combining data from various “omics” disciplines, is expanding at a fast pace [1]. A particularly promising combination is genomics linked with phenomics [2]. The latter approach is understood as a way to trace causal links between genotypes, environmental factors and phenotypes. This type of analysis is a complex task usually involving the handling of multidimensional datasets. In human health and plant science, complex and costly data collection and evaluation are frequently performed [3–6]. However, there has been difficulty in employing this strategy in the field of insect integrative biology [7].

Phenotypic plasticity describes the capacity of a single genotype to exhibit a variety of phenotypes as well as the mechanisms that translate environmental variation into reproducible phenotypic modifications [8]. If “phenotype” is understood in a broad sense, including all aspects of an organism other than the genotype, from the enzyme products of the genes to learned behaviors and the “environment” to include both the external surroundings of an organism and the internal conditions affecting gene expression, “phenotypic plasticity” is seen to encompass an enormous diversity of kinds of variability [9]. Distinction should be made between phenotypic plasticity and polyphenism, the latter being understood as the existence of environmentally cued alternative phenotypes in a population. Recently a comprehensive work has investigated the polyphenism and phenotypic plasticity in five related species of mycalesine butterflies from the Old World tropics, exhibiting seasonal polyphenism. For example, the differences between wet and dry season form of *Bicyclus anynana*, and those of many other mycalesines, are the color patterns on the ventral wing surfaces strongly associated with the alternative seasonal strategies to avoid predation. In the laboratory, the seasonal forms of *B*. *anynana* can be induced by the temperature control during the critical period of pre-adult development [10].

An interesting point is made in a focus paper tentatively applying IBM Watson to pathology: “Remember, from the whole genome sequence perspective, there is no difference between a caterpillar and a butterfly.” [3]. Indeed, despite exhibiting distinct body structures, diets, behavior, survival strategies, etc., the caterpillar and the butterfly represent the expression of the same genetic information. In a certain sense, they can be regarded as two different phenotypes of the same genetic information. The switchover between the larval form and the imaginal form takes place in the pupa, which takes only slightly longer than one week in many species. Furthermore, as concisely formulated by Nijhout: “With (genetic) dominance, a particular phenotype can be produced by several different genotypes. The uncertainty works in the other direction as well: a single genotype can produce many different phenotypes, depending on various contingencies encountered during development. That is, the phenotype is the outcome of a complex series of developmental processes that are influenced by environmental factors as well as by genes” [11]. It is worth noting that for insects with complete metamorphosis (for butterflies in particular), there is an opportunity to induce species-specific changes in the pupal stage–when transformation from larva to imagine takes place–at a well-defined developmental time [12–14]. Some of these phenotypic changes induced by targeted interventions can be perceived by the naked eye. For example, we recently showed that for *Polyommatus icarus* butterflies (both males and females), the extent of the induced phenotypic alterations in pigment-based patterns on the ventral wing surface is proportional to the duration of cooling applied to the pupa immediately after pupation [14].

We also found that the blue structural coloration of the dorsal wing surface of *P*. *icarus* males showed a much smaller magnitude of change upon cooling than the pigmented pattern on the ventral wing surfaces [14]. The observed change was not proportional to the duration of cooling, indicating that only hidden genetic variations were revealed by the stress [15,16]. In contrast to pigment-based colors, structural colors arise from particular interactions between chitin-based nanoarchitectures in wing scales and electromagnetic radiation [17]. These nanoarchitectures are self-assembled during scale formation. Despite some insight to these special nanoarchitectures [18–22], their formation is not yet fully understood, nor is the mechanism that causes *P*. *icarus* males to exhibit structurally colored blue dorsal cover scales while females exhibit brown dorsal cover scales colored by pigment.

In the case of structural colors, the characteristic dimensions–in the tens of nanometers range–and the periodicity of the nanoarchitecture determine which wavelength ranges cannot propagate in the nanostructure and are reflected towards the eye of the observer [17]. Changes of the characteristic nanostructures in the range of a few tens of nanometers can produce a significant shift in the spectral reflectance maximum [23].

Surprisingly, all cooled *P*. *icarus* females exhibit a number of blue scales with a similar structure in their lumen to that of the males [14]. The number of these scales varies from individual to individual, with the general trend of longer cooling yielding an increasing number of blue scales. In natural populations in Central Europe, females exhibit pigment-based brown coloration on the dorsal wing surface, and the frequency of bluish females in museum collections is usually below 10% [14]. Here, one must take into account that collectors tend to keep those individuals that are “unusual”; therefore, bluish females are overrepresented in these collections.

As discussed in detail by Houle et al. [2], the multiple ways in which genotypic information influences the phenotype of an organism can be best represented in a genotype-phenotype (G-P) map (Box 1 in [2]). This approach extends back to Richard Levontin’s work stating that evolution takes place in the space of all possible genotypes (G space) and the space of all possible phenotypes (P space) [24]. A central point under this approach is the decomposition of evolution into two processes taking place in two distinct “spaces”: G space and P space. There are four key components of this evolutionary process: (1) the epigenetic process generates the phenotype using genotypic information; (2) natural selection acts in P space to shift the average phenotype of the parents away from the average phenotype of all individuals; (3) the identity of successful parents determines which genotypes are preserved; and (4) genetic processes such as mutation and recombination alter the position in G space [2]. Convincing examples of this process are provided by the numerous varieties of fruits, such as apples, and of dogs, cats and other domestic animals that have been generated by targeted human selection from essentially the same genotypes, which has been achieved in only the last two thousand years in many cases.

Under the above approach, it is tempting to examine whether a given “environmental factor”, such as prolonged cooling of the pupae, may have reproducible effects on the resulting phenotypes of a certain genotype. Our test species of butterfly, *P*. *icarus*, is particularly well suited for this type of experiment, as the blue structural coloration on the dorsal wing surface of this species used for sexual communication [23] exhibits a high degree of stability [14,25], while the alteration of the pigment-based pattern on the ventral wing surface exhibits fairly linear dependence on the duration of cooling at 5 °C to which freshly formed pupae are subjected. Therefore, we repeated our earlier cooling experiments using 200 pupae resulting from 220 larvae reared under controlled conditions as described previously [14].

To get further insights in the phenotypic modification induced by prolonged cooling of the freshly formed pupae of *P*. *icarus* butterflies [14] we wanted to answer a number of questions:

- The linear dependence and the slope, characterizing quantitatively the magnitude of the modifications on the ventral wing patterns versus the cooling time, is the same between different experiments?
- Our previous results indicated that the genes governing the production of the photonic nanoarchitecture responsible for the blue coloration of the dorsal wing surface of the males must be present in the genome of both sexes [14]. Linked to this: if all the progenitors originate from the same population, and were collected at the same time, how large is the deviation in the position of the blue reflectance maxima of the females with induced blue coloration?
- Is there a limit in the number of brown pigmented scales converted to blue colored by nanoarchitectures, or is it possible to convert most, or all? This conversion process implies a fundamental modification of the cellular processes as the two coloration mechanisms are radically different.
- The common origin and the number of the reared larvae together were sufficient to allow the investigation of the relation of eclosed individuals and that of individuals eclosed with defects. This aspect was not discussed in our previous work.

We found that the alteration of the pigment-based pattern of the ventral wings induced by controlled cooling exhibited a remarkably high degree of reproducibility. Additionally, the blue coloration on the dorsal wings of the cooled females showed significantly greater deviation from the color of the wild males than did the spectral deviation of the cooled males. The fraction of butterflies that eclosed from cooled pupae showing defects after the termination of cooling indicated three stages in the duration of cooling: 0 to 4 weeks, 4 to 8 weeks, and 8 to 12 weeks. The changes in the arrangement of the scales and the micron-scale morphology of the blue scales of females support this division of the cooling time into three stages.

## Materials and methods

### Capture of imagines and egg laying

Wild first-brood *P*. *icarus* individuals were captured in the vicinity of the city of Érd (Hungary) (47°22´35.947˝N 18°51´18.55˝E) in the early spring of 2016. The species *P*. *icarus* is not subjected to any restriction in Hungary. All samples were collected on public land that was not protected in any way and for which no specific permissions were required. We placed three females and three males in cages containing a mixture of food plants for these larvae (*Trifolium pratense*, *Trifolium repens*, and *Medicago sativa*). The cages were situated in a garden-type environment as reported previously [14]. The imagines were fed with a sugar-water solution. The mating of females and males was observed inside the cages. The females laid eggs on the food plants in the cage, exhibiting a strong preference for *M*. *sativa* in the sunniest areas of the cage. The period of egg laying varied from 10–14 days.

### Larvae rearing in cages and in the laboratory

After hatching from eggs, the small larvae were allowed to feed freely in the cage until the condition of the food plants started to decline at an accentuated pace. At this stage, the plants were cut close to ground level and taken to the laboratory, where the larvae were collected. The larvae were in different stages of development depending on the time at which the egg was laid. Each larva was placed in a separate Petri dish, which was cleaned daily, and the larva was given fresh food plant leaves. The larvae were fed until the characteristic prepupa stage was observed, usually at one to two days before pupation. Following pupation, the freshly formed pupae were transferred to clean Petri dishes with plant leaves (for the stabilization of the humidity), which were then placed in a refrigerator in the dark at a constant temperature of 5 °C.

### Cooling following pupation

The developmental stage immediately after pupation is well defined and was therefore chosen as the starting point for cooling. The duration of cooling was set on the basis of previous experiments [14]. Six cooling times were chosen: 2 weeks, 4 weeks, 6 weeks, 8 weeks, 10 weeks, and 12 weeks. For each cooling period, a group of 30 pupae was used, and a few pupae were kept in reserve for those cases in which a cooled pupa did not survive cooling for some reason. Most frequently, these cases involved pupae that were collected too late after pupation in which ecdysone production reached the threshold [11,26] at which the development of the pupa could not be stopped, even when placed in the refrigerator for cooling to 5 °C.

### Resumption of pupal development after cooling for a long duration

In the absence of cooling, *P*. *icarus* pupae undergo a gradual color change from green to light yellow, dark yellow, light brown and, finally, dark brown over 8–10 days. One day before eclosion, when the pupal cuticle hardens and becomes transparent, blue coloration of the males and orange spots on the female dorsal forewings can already be observed inside the pupa in most cases.

After the planned cooling time had elapsed, the pupae were removed from the refrigerator and placed in the laboratory in closed transparent plastic boxes, which allowed exposure to normal daylight conditions. The humidity level in the boxes was regulated by a free water surface. The pupae were isolated from the water container by using a net, which was also used to allow the eclosed butterflies to climb to the top section of the box to extend their wings. The adult butterflies that successfully eclosed emerged 8–10 days after the pupae were removed from the refrigerator.

A single group of cooled butterflies, in which the butterflies were cooled for 4 weeks, was treated in a different way. In this case, the box in which the postcooling development of the pupae was carried out was placed far away from the windows, where only weak illumination was provided during the daytime, to test the effect of light conditions on pupal development.

### Physical evaluation

#### Photography of both wing surfaces

Photographs were taken using a Canon EOS 5D Mark III (Tokyo, Japan) digital camera. To ensure proper comparisons, we used the same illumination conditions employing the same halogen light source in every image.

#### Micrography of wings and single scales

Micrographs of wings under reflected light and scales under reflected and transmitted light were taken using a Zeiss AxioImager A1 (Jena, Germany) microscope with an attached digital camera.

#### Measurement of spectra on single scales removed from the wings

Transmittance and reflectance in the 400–700 nm wavelength range were measured on single scales with an Avantes Avaspec-HS1024 TEC (Apeldoorn, Netherlands) spectrometer attached to a Zeiss AxioImager A1 microscope. The scales were placed on glass microscope slides, which also served as a reference in the calculation of the proportions of reflected and transmitted light.

#### Spectral measurements on the dorsal wing surface and location of reflectance maxima

Spectral measurements were conducted using an Avantes deuterium-halogen light source (AvaLight DH-S-BAL) and an Avantes Avaspec-HS1024 TEC UV-VIS-NIR modular spectrophotometer. This setup provided illumination and allowed detection in the 200–1100 nm wavelength range. The collection of reflected light was performed using an integrating sphere, where a 5 mm spot was illuminated perpendicular to the wing surface and the light reflected at all angles to the upper hemisphere was collected [25]. In all measurements, a white diffuse standard tile (Avantes WS-2) was used as a reference. For some specimens, normal-incidence spectral measurements were also conducted. In this case, the Avantes normal-incidence reflection probe was used (Avantes FCR-7UV200-2-ME-SR), in which the illuminating and detecting optical fibers were combined in one probe, allowing us to detect the perpendicularly reflected light from the wing surface.

In the specimens in which a sufficient number of blue scales were found, a significant peak in the near-UV-blue wavelength range could be observed. For further analysis, these peaks were characterized using OriginLab Origin 2018 (Northampton, MA, USA) software, and the wavelength and intensity values of the peaks were determined.

#### Electron microscopy

Scanning electron microscopy (SEM) was conducted using an LEO 1540XB (Zeiss GMBH, Jena, Germany) microscope. The butterfly wings were cut into pieces of a few mm^2^, which were then attached to a sample holder that allowed us to inspect the desired regions of the wing surfaces. To preserve the original condition of the wing scale nanoarchitectures, we did not apply metallic coating.

Transmission electron microscopy (TEM) was preceded by preparation of the samples. After fixing (45 min in 2.5% glutaraldehyde and 2% formaldehyde in 0.1 M Na-cacodylate buffer (pH 7.2)) and dehydration (in graded ethanol series (50–100% in 10% steps)), few millimeters of wing pieces were embedded in Spurr’s resin (SPI Supplies, West Chester, PA, USA) and were stained with uranyl acetate and lead citrate to enhance the contrast of the prepared images. Using an ultramicrotome, 70-nm-thick slices were cut and placed on TEM microgrids. Samples produced in this way were investigated using a Philips CM20 (Philips, Eindhoven, The Netherlands) microscope at an accelerating voltage of 200 keV.

The thicknesses of the chitinous and air layers were measured in the TEM images using CorelDRAW X6 (Corel, Ottawa, Ontario, Canada) software. The SEM images were analyzed using our custom-made BioPhot Analyzer 2.0 software [27] which allowed us to extract the structural parameters of the investigated pepper-pot-type structures.

#### Evaluation of alterations of the pattern on ventral wing surfaces

The ventral sides of the wing surfaces of males and females exhibit similar complex pigmentation, consisting of basal, medial, postmedial, and submarginal patterns of black spots with white rings and submarginal orange lunules, which are typical of the subtribe Polyommatina. It is possible to assign a numerical value to any deviation from the standard pattern observed in normal individuals, allowing quantitative evaluation of the degree of aberration. We qualified and coded wing size and the ventral wing-surface coloration and pattern in comparison with individuals of the control group as a standard. The numerical values of the aberrations were averaged for butterflies eclosed from pupae that had been cooled for the same number of days. The quantification procedure is described in detail in [14].

#### Statistical analysis

The statistical analyses were carried out using Origin 2018 (OriginLab, Northampton, MA, USA) software. The raw data of the samples was evaluated using One-way ANOVA supplemented with post hoc Tukey’s test. For the comparison of the linear regressions F-test was conducted.

## Results

### Reproducibility of cooling-induced changes in the pigment-based pattern

**Fig 1:**
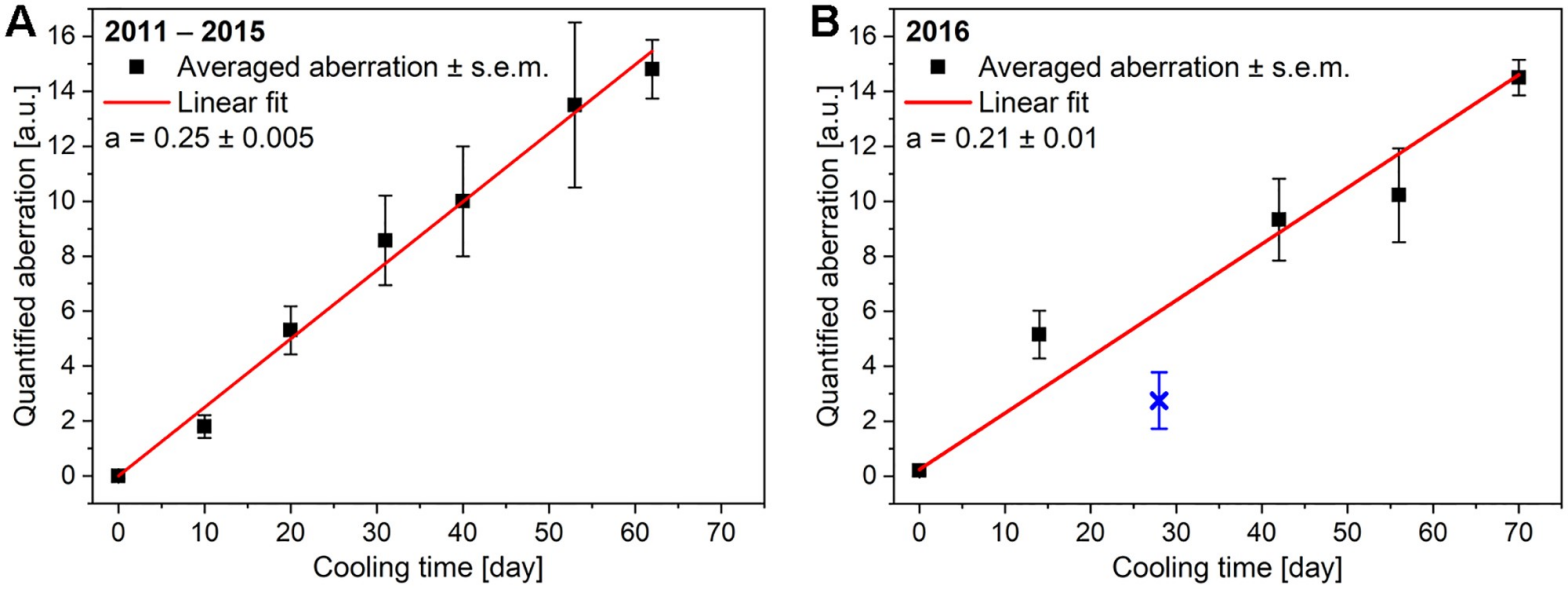
Quantification of the aberration of the pigment-based pattern on the ventral wing surfaces of *P*. *icarus* butterflies. (A) Previously reported results [14]; (B) results of the set of cooling experiments conducted to check reproducibility. The vertical bars at each data point indicate the magnitude of the phenotypic differences between the individuals within a group cooled for a given duration. (s.e.m. = standard error of the mean).

The results of the two series of cooling experiments–the results published previously [14] and those of the present work–carried out on freshly formed pupae of *P*. *icarus* butterflies are shown in Fig 1. Comparison of the two graphs shown in Fig 1 reveals a high degree of reproducibility in the results. The statistical analysis showed no significant differences between the two datasets (F-test, p = 0.0971) (S1 Fig). Additionally, the second set of experiments is characterized by much smaller error bars than the first set. This difference may be attributed to the progenitors originating from the same population and the larger number of pupae used for each cooling time in the second set of experiments.

S2 Fig shows some of the pupae after 10 weeks of cooling, whose appearance does not differ from the appearance of freshly formed pupae. Thus, these pupae had not yet undergone the typical color changes that can be observed during pupal development. This finding indicates that their development was indeed “frozen” in the stage following pupation. When the cooled pupae were brought to room temperature, they followed the normal color change sequence, from yellowish-green to yellow, brown, and, finally, blue in the case of males or dark brown in females. The blue coloration of the male pupae is usually observed one day prior to eclosion. Its presence indicates that the photonic nanoarchitecture has formed in the scales and that the nanovoids within this architecture are filled with air. If they were instead filled with body liquid, or even pure water, the structural color would not be visible, as the refractive index contrast between chitin (n = 1.56) and water (n = 1.33) would be insufficient [28].

**Fig 2:**
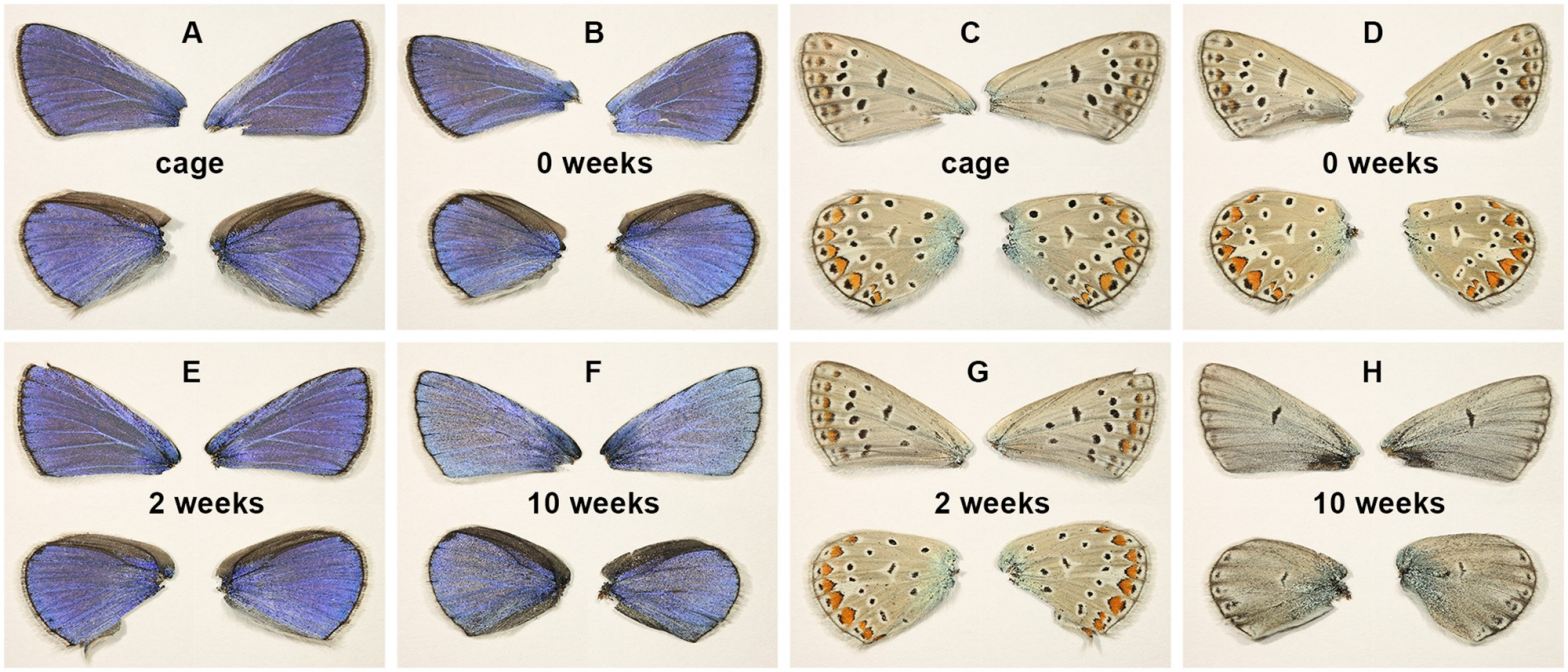
Dorsal and ventral wing surfaces of *P*. *icarus* males. (A,C) Specimens reared in the cage; (B,D) reared in the laboratory without cooling; or reared with cooling of the pupae for (E,G) 2 weeks or (F,H) 10 weeks.

In Fig 2, the four dorsal and four ventral wing surfaces are shown for male *P*. *icarus* butterflies eclosed from pupae maintained in the cages in the garden and allowed to complete their transformation there; for pupae that underwent transformation in the laboratory without cooling; and for pupae after 2 weeks, or 10 weeks of cooling. While the structural blue coloration of the males shows small changes after 10 weeks of cooling (mainly in the saturation of the blue color), the pigment-based pattern has completely lost almost all characteristic elements of the species, some of which are still observable after 6 weeks of cooling.

### Individual variations in pattern changes

While the average ventral pattern changes under a certain cooling time exhibited good reproducibility between experiments carried out in different years, there could be differences within a group of individuals cooled for a certain duration. To illustrate this, we chose the group cooled for 6 weeks because it exhibited the greatest magnitude of differences between individuals and was composed of three males and nine females eclosed in such a way that their wings could be evaluated. In S3 Fig, it can be seen that the changes can be roughly divided into two groups: the first group is similar to what is shown in Fig 2, with only traces of the typical pattern for the wing margins being visible, while the second group is similar to what is shown in Fig 1L and Fig 1P of [14], with some of the black postmedial spots surrounded by white halos also being preserved. Among the nine female specimens, three belong to the second group and six to the first group.

The increase in the length of the cooling period produced a shift towards individuals in which most elements of the pigment-based pattern on the ventral wing surface were lost, as seen in the male in Fig 2 subjected to 10 weeks of cooling. In fact, after 10 weeks of cooling, all the individuals that eclosed with wings allowing pattern evaluation exhibited this type of excessively reduced pattern.

All the cooled females–starting with those subjected to 2 weeks of cooling (i.e., all 16 individuals)–exhibited some blue scales, but the number of blue scales varied from only a few to many, as shown in S4 Fig.

**Fig 3:**
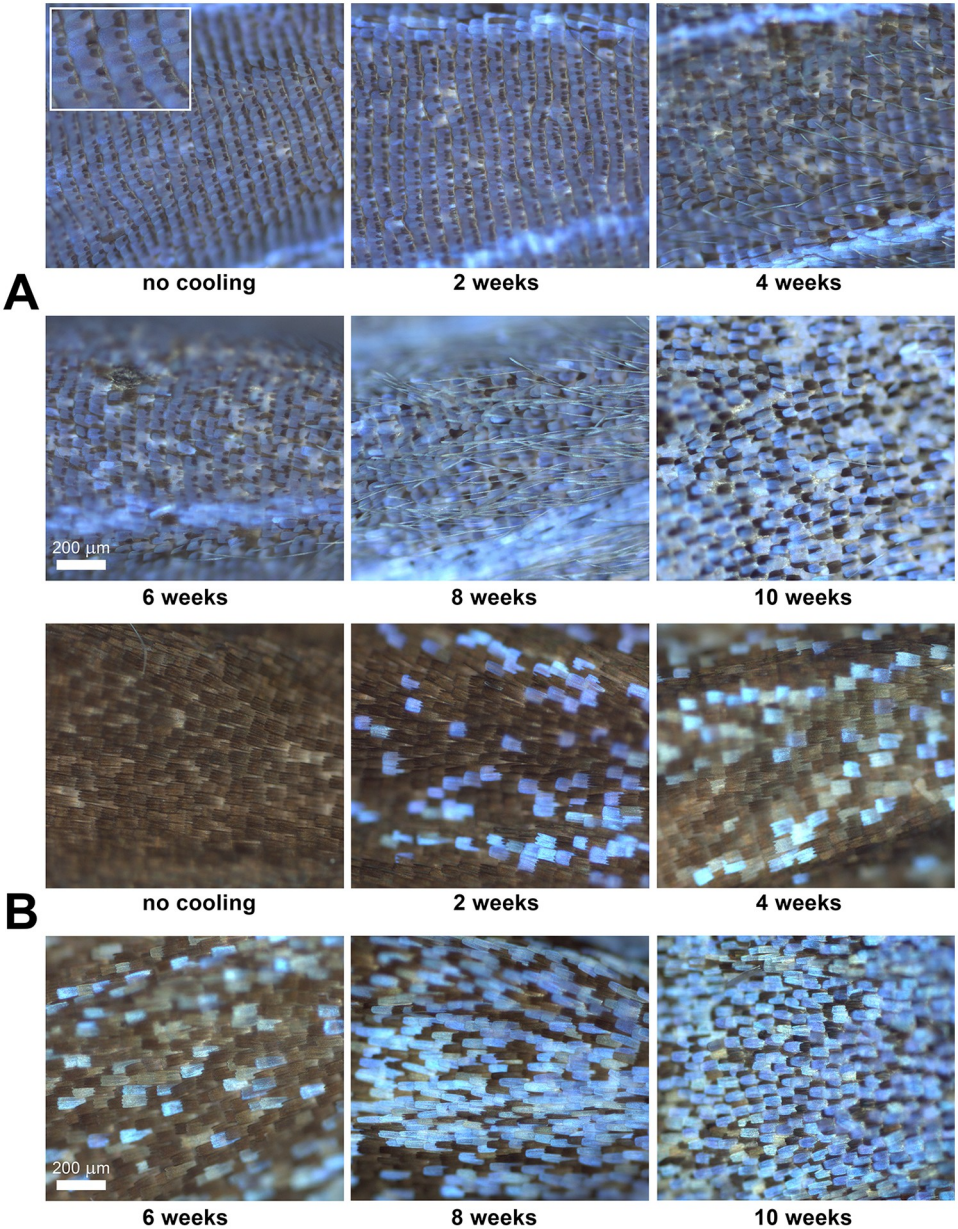
Changes in the dorsal wing scales of *P*. *icarus* specimens with increasing cooling times, as seen under an optical microscope. (A) Male and (B) female specimens. The inset in the top left corner of (A) shows the androconia separating the regular rows of blue cover scales.

**Fig 4:**
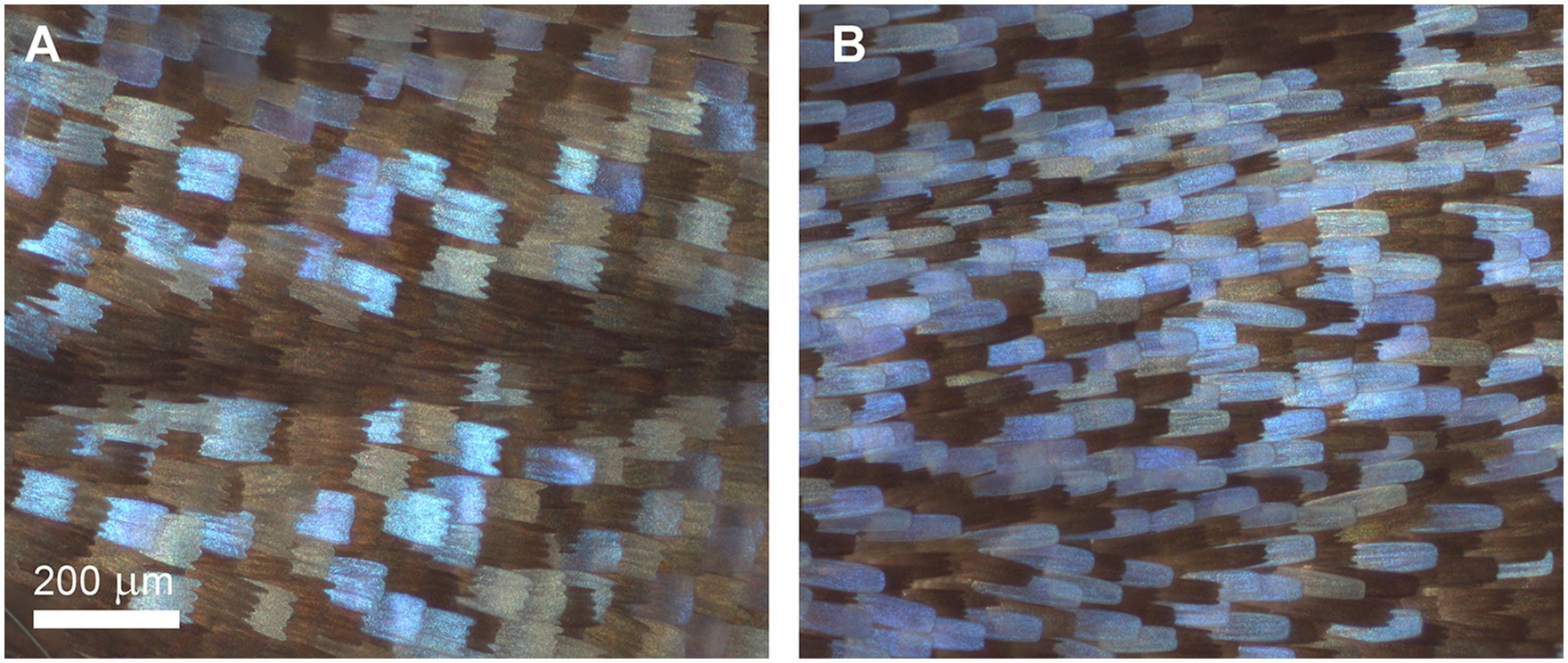
Detailed comparison of the morphology of blue scales of female *P*. *icarus* specimens. The pupae were cooled for (A) 4 weeks and (B) 8 weeks.

The gradual changes under increasing cooling times can be observed in the optical micrographs presented in Fig 3. The morphological changes of the dorsal wing scales of female specimens after 4 and 8 weeks of cooling are presented in Fig 4.

**Fig 5:**
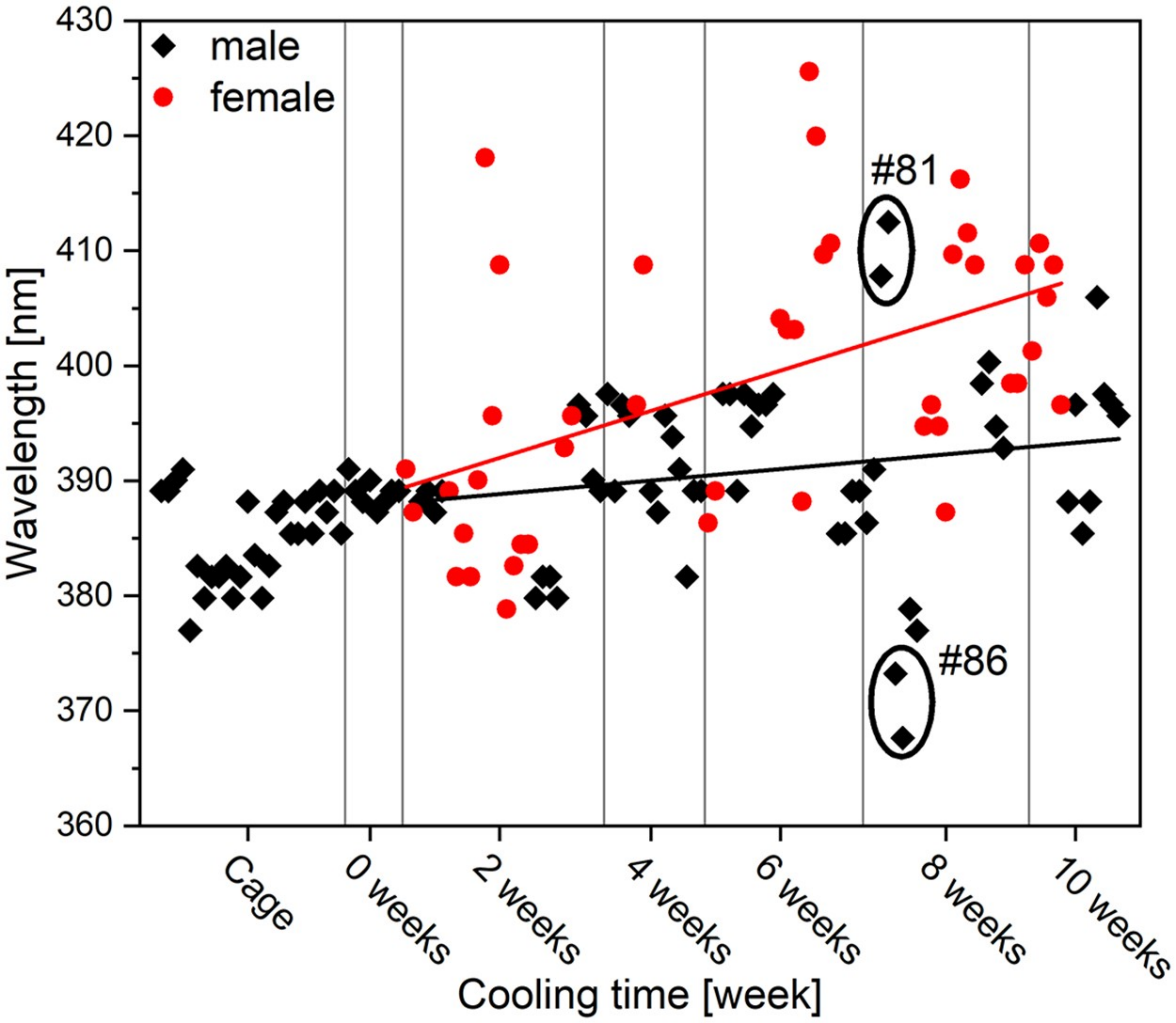
Spectral position of the blue maximum measured on the wings of *P*. *icarus* butterflies eclosed from cooled pupae. Continuous lines were drawn according to the linear fit of the black (red) symbols.

The variation in the coloration of individual wings can be best presented using the spectral position of the blue maximum measured on the dorsal wing surfaces. All males whose wings were flat enough to allow spectral measurements were included in this analysis, whereas only those females that exhibited a sufficient number of blue scales to allow unambiguous determination of the reflectance maximum were included. The results are shown in Fig 5. When possible, all four wings were subjected to measurement and are presented in the figure as separate objects. The macroscopic color of the wing is a result of the average color of the microscopic scales, and each wing is therefore considered as an individual optical object. In certain cases, the crumpling of the wings after eclosion did not allow the measurement of all four wings.

As shown in Fig 5, with the increase in the cooling time of the pupae, the black symbols around the line drawn according to the linear fit to the spectral position of the blue maxima of the males exhibit only moderate deviation (covering only 10 nm) including the 6-weeks-of-cooling group, and the continuous line deviates only very slightly from the horizontal line defined by the wild and the uncooled males. On the other hand, the blue coloration of the females shows increased deviation after as little as 2 weeks of cooling (covering 35 nm), and the continuous red line in Fig 5 deviates significantly (F-test, p = 0.00492) from that of the males (black line).

**Fig 6:**
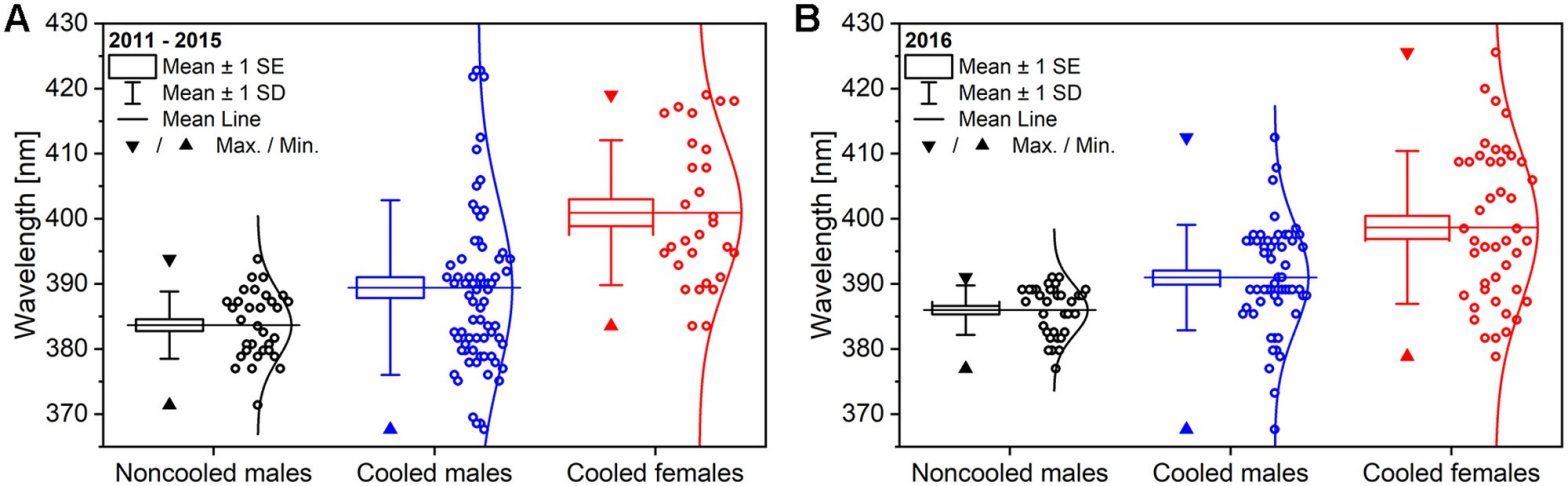
Box plots of peak wavelength of the dorsal blue coloration measured on *P*. *icarus* noncooled males, cooled males, and cooled females of the two experiments. Experiments from (A) 2011–2015 and (B) 2016 are shown. One-way ANOVA showed significant differences between the means (p < 0.05) and the post hoc Tukey-test showed significant differences between all three groups of samples.

The box plots of the peak wavelength of the dorsal blue coloration measured in the noncooled males, cooled males, and cooled females in the two experiments conducted in 2011–2015 and 2016 are shown in Fig 6. The two plots (n_2011-2015_ = 27, n_2016_ = 64) show remarkable similarities regarding the means, and the standard errors and deviations. One-way ANOVA was conducted and showed significant differences between the means of the noncooled males, cooled males, and cooled females in both experiments (p < 0.05) which was confirmed by post hoc Tukey-test for all pairs.

**Fig 7:**
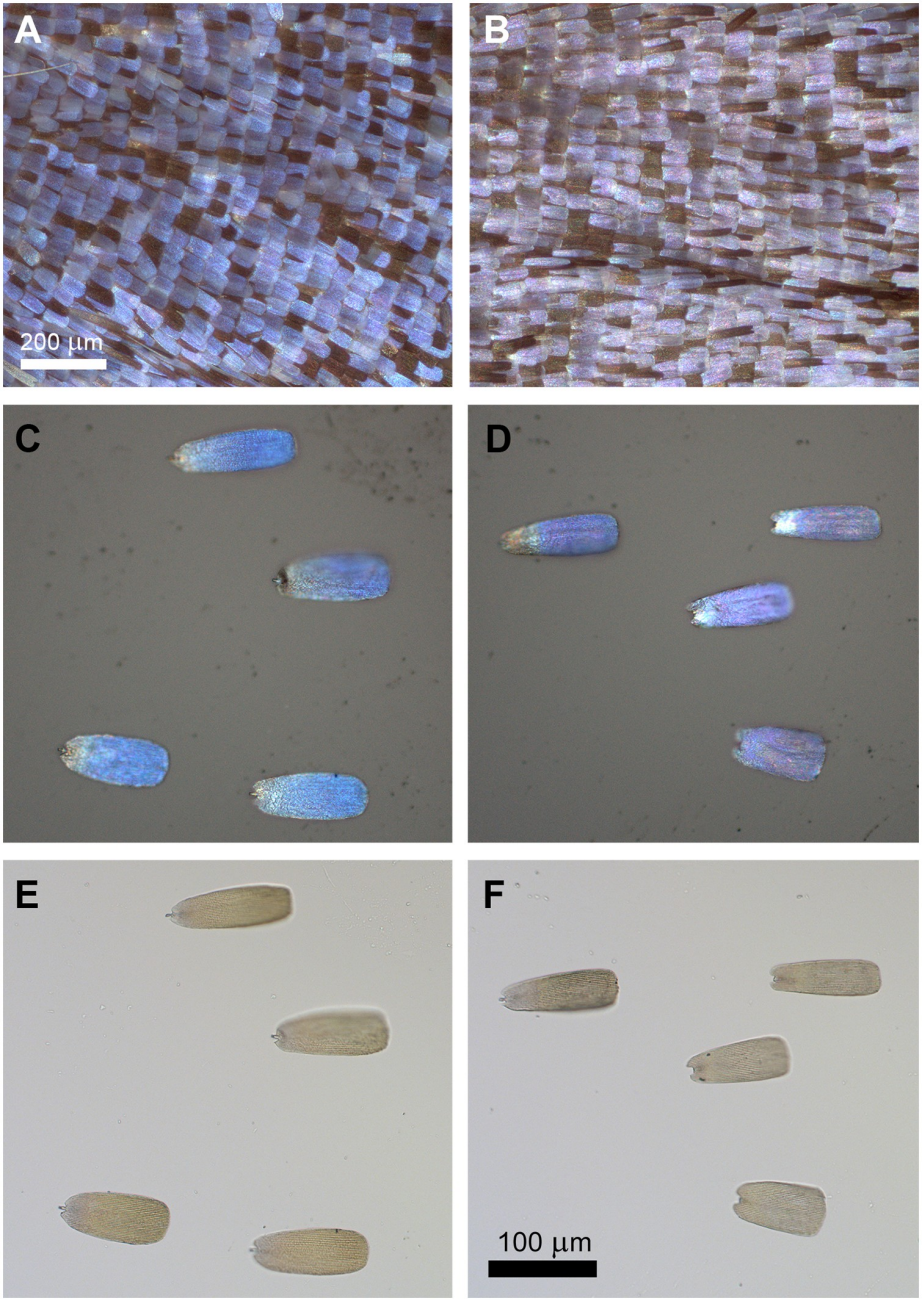
Optical micrographs of dorsal hindwings and individual scales of *P*. *icarus* males #81 and #86. The micrographs were acquired rigorously under the same illumination and magnification conditions. (A) Dorsal hindwing of #81 under reflected light; (B) dorsal hindwing of #86 under reflected light (C) #81 wing scales under reflected light; (D) #86 wing scales under reflected light; (E) #81 wing scales under transmitted light; (F) #86 wing scales under transmitted light.

We investigated the individual differences between the males eclosed after 8 weeks of cooling in detail. Males #81 and #86 were selected because they exhibited the largest differences in the spectral position of their blue reflectance maxima, which were 388 nm for male #86 and 416 nm for male #81. The optical micrographs of the dorsal hindwings and the separated individual wing scales of the two males are shown in Fig 7.

The ventral wing surfaces of these two males also exhibited significant differences, which are shown in S5 Fig. The wings show similar differences in their patterns to those presented in S3 Fig.

**Fig 8:**
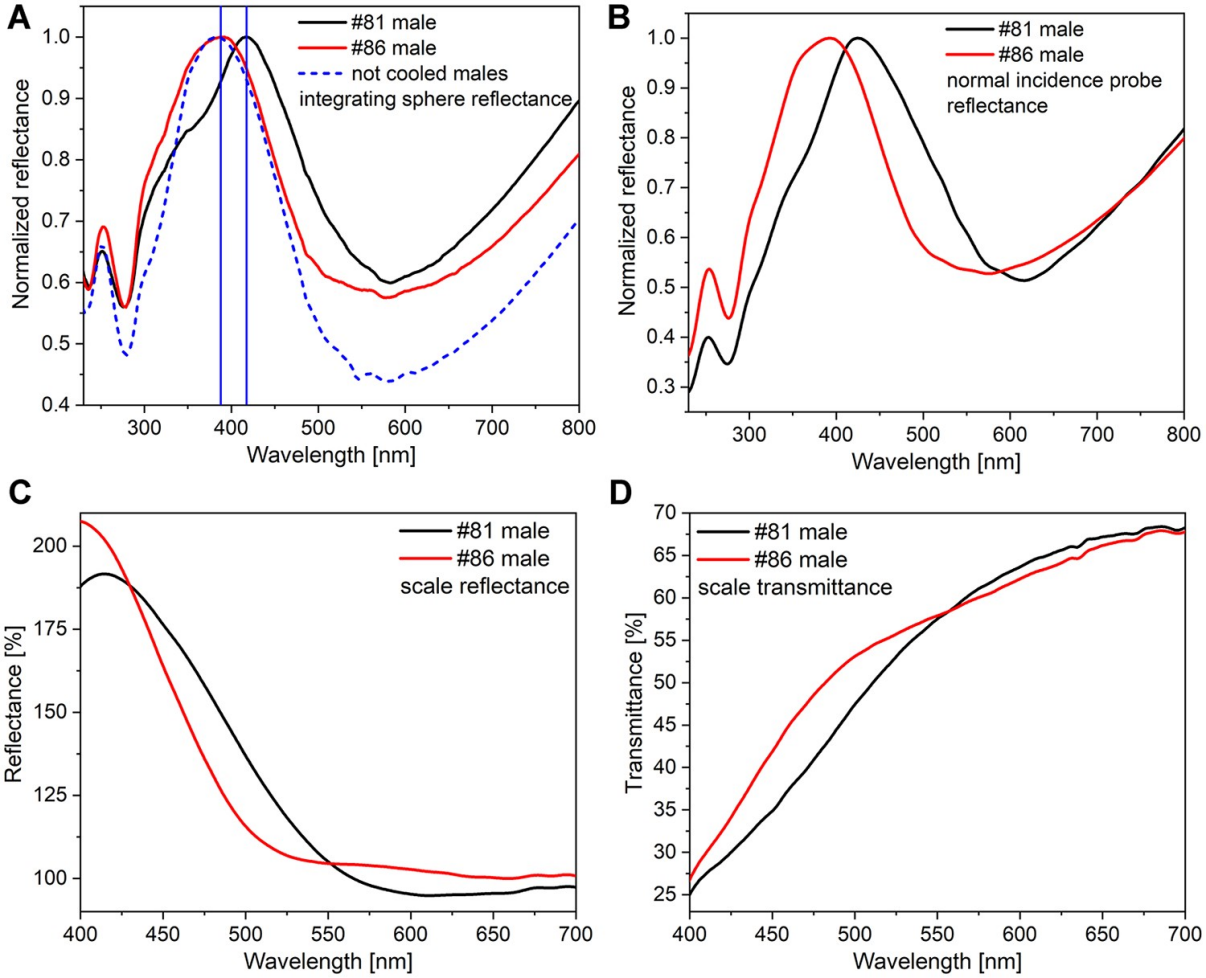
Reflectance spectra of *P*. *icarus* males #81 and #86 measured on whole wings with (A) the integrating sphere, and (B) the normal incidence probe; (C) reflectance and (D) transmittance spectra of the single scales of these specimens measured with the microspectrophotometer. In (A), the broken blue line indicates the spectrum resulting from averaging the reflectance of 7 noncooled exemplars. Averaged spectra (n = 4) measured with a microspectrophotometer on the individual scales shown in Fig 7: (C) reflected light; (D) transmitted light.

The reflectance spectra measured by both the integrating sphere and the normal incidence probe revealed clear differences in the spectra of the two males when the measurements were performed on whole wings (Fig 8A and 8B) or on individual scales by using a microspectrophotometer (Fig 8C and 8D). In the latter case, one must take into account that the limited transmittance of the optical elements under a standard optical microscope distorts the measurements in the UV range. However, the distortion is identical for the two samples, so the differences between the samples can be effectively revealed.

In Fig 8A and 8B, the reflectance of two males cooled for 8 weeks can be compared with the spectrum resulting from averaging the reflectance of males from the same experiment that eclosed from pupae that were not subjected to cooling. One may observe that the reflectance of male #86 and the average reflectance of the uncooled males are almost coincident in the blue range, while male #81 exhibits a markedly different shape.

**Fig 9:**
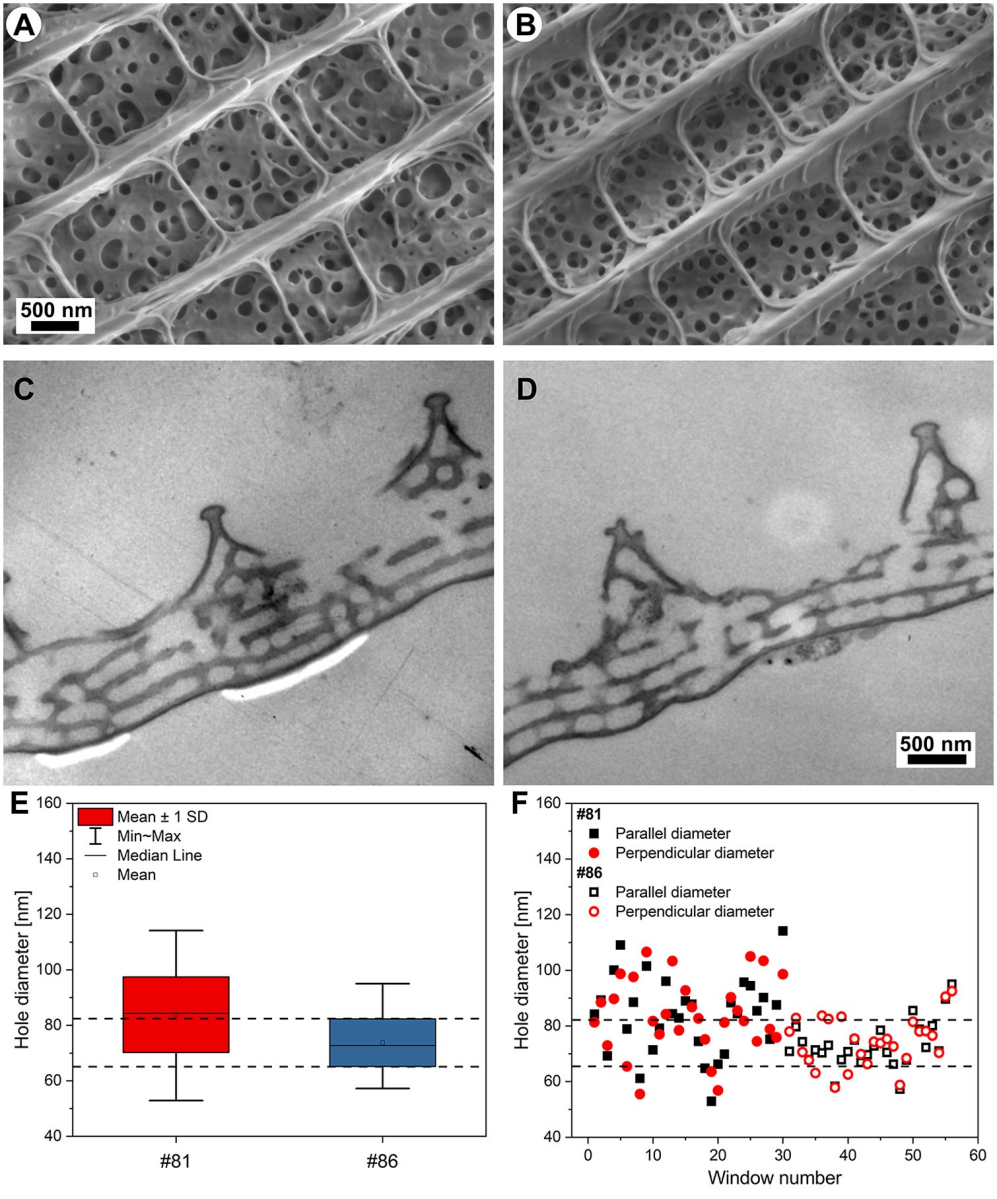
SEM, cross-sectional TEM images, and hole dimensions measured on the wing scales of *P*. *icarus* males emerged from pupae cooled for 8 weeks. The color-generating photonic nanoarchitectures of (A,C) male #81; (B,D) male #86 can be seen on (A,B) SEM and (C,D). TEM images. (E) Box plots and (F) the raw data of the structural analysis are shown. The One-way ANOVA showed significant differences (p < 0.001) between the hole diameters in the two specimens’ photonic nanoarchitectures.

SEM and cross sectional TEM images of the cover scales of male #81 and #86 specimens are shown in Fig 9. Detailed structural analysis of the wing scale nanoarchitectures of the two specimens was conducted based on 10 SEM and 6 TEM images. It was found that there are small but characteristic differences between the layer thicknesses of the multilayer structures (S1 Table) and statistically significant differences were found using One-way ANOVA (p < 0.001) between the average diameters of the perforated air holes in the photonic structure, which can lead to the spectral differences of the two specimens. Fig 9E and 9F shows the box plots and the raw data of the air hole diameters measured on the SEM images of the photonic nanoarchitectures.

### Proportion of eclosion and imperfections as a function of cooling time

Increasing the cooling time resulted in a decrease in the probability of defect-free eclosion. Defect-free eclosion means that all four wings were formed and spread out regularly.

The eclosed fraction was calculated as the ratio of eclosed individuals to the total number of pupae cooled for a certain time (30 pupae for each cooling time) in the present study. The proportion with defects was calculated as the ratio of the number of imperfectly eclosed individuals to the total number of eclosed individuals.

**Fig 10:**
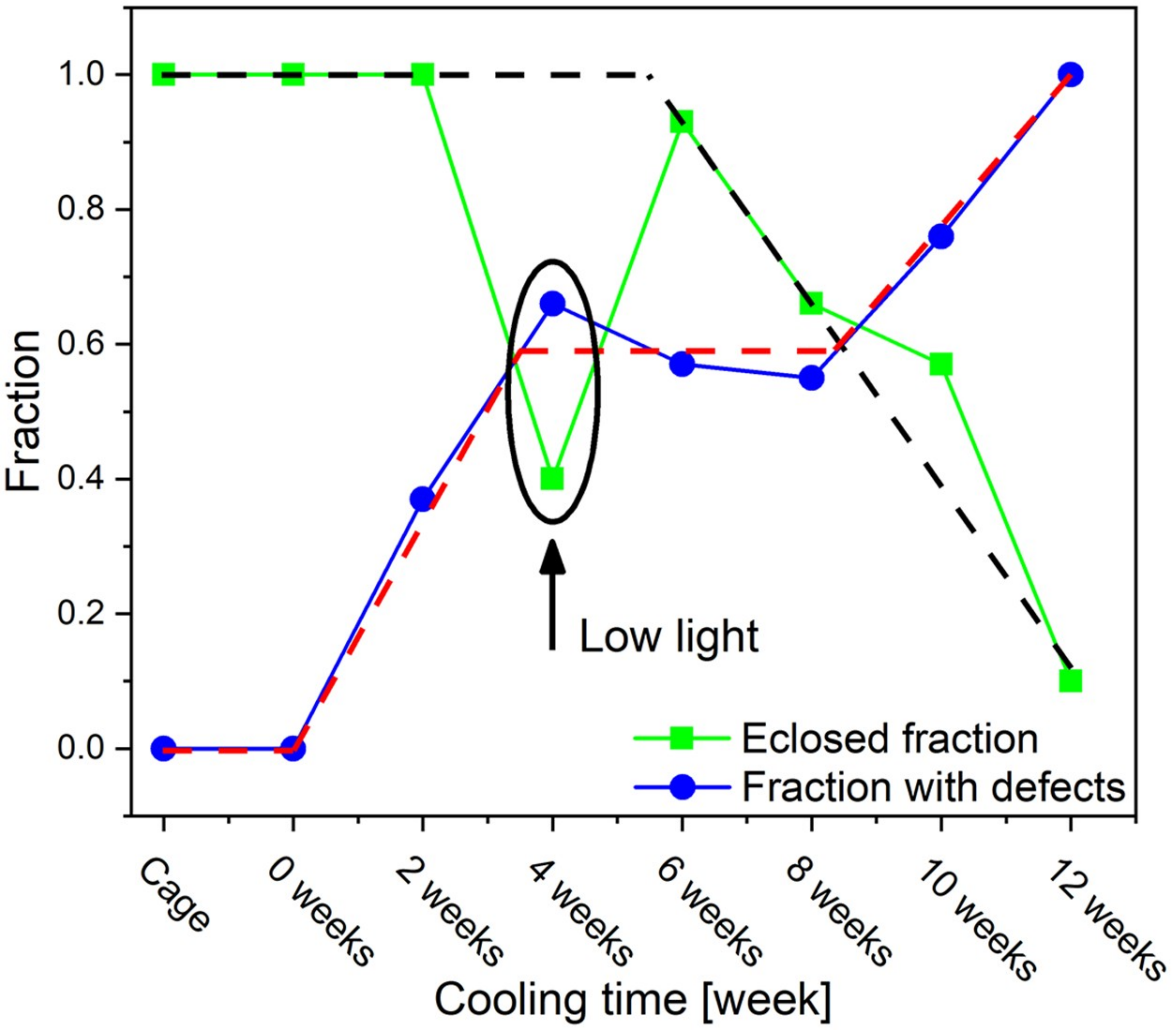
Fraction of eclosed (green line) *P*. *icarus* butterflies and of butterflies that eclosed with defects (blue line) as a function of the cooling time applied to the pupae. The broken black and red lines were drawn to guide the eye. The pupae that were cooled for 4 weeks were allowed to resume development after cooling under low-light conditions.

As shown in Fig 10, the eclosed fraction and the fraction with defects exhibited differences in the dependence on cooling time: while two weeks of cooling did not decrease the eclosed fraction, this cooling time yielded a number of eclosed butterflies with imperfections (11 butterflies from 30 pupae), most frequently in the form of imperfectly spread wings. The shape of the black broken line suggests that the eclosed fraction started to decrease sharply at between 4 and 6 weeks of cooling, while the number of exemplars that eclosed but with defects exhibited a plateau between 4 and 10 weeks of cooling. After 12 weeks of cooling, no defect-free eclosion was observed.

From the shape of the green curve in Fig 10, it follows that low-light conditions during the resumption of pupal development were very detrimental to the number of eclosed butterflies (12 imagines from 30 pupae) and were likely to have slightly increased the fraction of individuals that eclosed with imperfections (8 of 12 individuals). It can be seen in S6 Fig that the pupae that were cooled for 4 weeks and failed to achieve eclosion underwent the expected color changes from green to brown (as detailed above) but failed to complete the developmental cycle.

As shown in Fig 1B, the low-light conditions also seemed to cause deviation of the point at which the pigment-based pattern on the ventral wing surface diverged from the general trend defined by the other points determined from experiments during which normal daylight, but not direct sunlight, was allowed during the resumption of pupal development.

### Emergence of pupae collected too late in the refrigerator

Those pupae that were subjected to cooling after the ecdysteroid threshold was reached (i.e., too much time elapsed between the formation of the pupa and the initiation of cooling in the dark) continued their development despite the temperature of 5 °C and the continuously dark environment–albeit at a much slower pace than under natural conditions–and reached various stages of unsuccessful ecdysis. Even under these conditions, these individuals reached the adult stage and attempted eclosion. However, no defect-free individuals were found among these individuals; some examples are shown in S7 Fig. Their development until eclosion took approximately 7 weeks instead of the normal 8–10 days. The development of the pupae in which cooling was initiated before this threshold was reached could be suspended for as long as 10 weeks with a few successful eclosions still occurring. The normal duration of pupation in *P*. *icarus*, depending on environmental conditions, is 7 to 14 days.

## Discussion

Polyphenisms are discrete alternative phenotypes that develop in response to specific signals from the environment [10,29]. Under natural conditions, different phenotypes usually develop in different periods of the year and are caused by differences in photoperiod, temperature, or nutrition [30]. The change from one phenotype to the other is mediated by a developmental hormone [31]. The principal hormones that control polyphenic development are the same ones that control molting and metamorphosis, namely, ecdysone and juvenile hormone. The external characteristics of insects are based on their cuticle; therefore, the expression of polyphenism requires a molt, and the alternative phenotype is expressed in the new cuticle that is synthesized during the molt. Prior to this molt, there are one or more relatively brief periods during which hormones can alter the course of subsequent development. These are called critical periods or hormone-sensitive periods, and the mechanisms involved act essentially as binary switches, with alternative developmental pathways being selected depending on whether a hormone is above or below a threshold value [28,30]. Most known cases of polyphenic switching of developmental pathways result from environmentally induced changes in the timing of hormone secretion, the timing of a hormone-sensitive period, or the threshold of hormone sensitivity [32]. Some other mechanisms have also been proposed more recently [33].

### Cooling-induced changes in the pigment-based pattern

The artificial conditions applied in our experiments (i.e., the unusually long duration of the cold period and the absence of light) corresponded to non-natural conditions that are not normally encountered by *P*. *icarus* during its development, as this species adapts to cooler climates by reducing the number of broods to a single brood in northern regions [34]. The shortest duration of cooling (two weeks) induced relatively minor changes in the pigment-based pattern (see Fig 2) and did not reduce the probability of eclosion but did increase the number of individuals that did not eclose in perfect condition to almost 40%. These changes may be associated with the perturbation of the hormone synthesis sequence immediately after pupation. The relatively good tolerance of two weeks of cooling may indicate that periods of this length in which climatic conditions are not favorable may be experienced by the first brood of *P*. *icarus* in spring from time to time in natural habitats. On the other hand, a five-times longer cooling time of 10 weeks induced dramatic changes in the ventral wing pattern, while leaving the structural color of the dorsal wing surface of males almost unchanged. Some observed disordering of the scale arrangement (Fig 3) will be discussed in more detail below.

Notably, not all pigment synthesis processes were affected in the same way. Both females and males exhibited a relatively unmodified “background color” on the ventral wing surfaces, as shown in Fig 2. The changes in the hue of this background color were close to negligible compared with the changes in the characteristic pattern of dots. Moreover, the sexual differences in this coloration were preserved, as the males presented a grayer background color, while the females presented somewhat browner coloration on the ventral wing. On the other hand, the small black spots encircled by white rings typical of the postbasal and postmedial areas disappeared. The brown coloration on the dorsal wing surface of the females was preserved to a good extent, similar to what was reported in Fig 1 of [14]. Notably, not all small black features disappeared. Even after 10 weeks of cooling, the discal lines were still present on the ventral wing surfaces, as shown in Fig 2.

The high degree of reproducibility in the average changes in ventral wing pattern elements (Fig 1) is surprising and indicates that such experiments may shed light on the mechanisms governing the formation of these patterns. However, within a group cooled for a given duration, one may find individuals that present different degrees of changes in their ventral wing patterns; these differences can be tentatively associated with genetic differences. As shown in S3 Fig, two distinct groups were observed: one in which cooling (for 6 weeks) induced large changes in the ventral pattern (butterflies 2, 3, 5, 7, 8, 9, 10, 11, and 12) and another group in which the pattern was almost completely unaltered (butterflies 1, 4, and 6). Further genetic investigations may be needed to reveal the basis of these differences. It is worth emphasizing that all of the above individuals were subjected to the same conditions during the experiment; thus, their different phenotypes cannot be attributed to environmental or dietary factors but to different responses to the same type of stress. On the other hand, in the absence of this stress, the two (very likely different) genotypes result in a similar phenotype.

### Cooling-induced changes at the wing scale level

In the optical micrographs of the dorsal wing surfaces of males (Fig 3A), one may observe that the first two micrographs, corresponding to no cooling or 2 weeks of cooling, are very similar in appearance, and the cover scales are arranged in regular rows separated by rows of androconia. These results again show that 2 weeks of cooling is within the “tolerance limit” of this butterfly species. On the other hand, starting from 4 weeks of cooling, disordering of the scale rows starts to be observable, where the rows are no longer well defined. After 6 weeks of cooling, the rows can barely be distinguished, and after 8 or 10 weeks of cooling, one cannot observe clear rows in the arrangement of the scales (Fig 3A), in S8 Fig, the disordering of the scale sockets after 10 weeks of cooling is shown.

A similar observation can be made for females with respect to the number of blue scales (Fig 3B). In the case of females, the rows of scales are less well defined, even for the specimens that were not cooled. The fraction of blue scales increases greatly after 8 weeks of cooling (Fig 3B). A further observation can be made from detailed comparison of the morphology of the blue scales of females after 4 or 8 weeks of cooling. As shown in Fig 4, clear morphological differences are present: after 4 weeks of cooling, the morphology of the blue scales is relatively unchanged, with the scales exhibiting dentate endings similar to the nearby brown scales; however, after 8 weeks of cooling, the blue scales exhibit a more elongated shape and rounded ends. The dorsal cover scales of wild and uncooled females differ markedly from the dorsal cover scales of males in terms of color (males: blue, females: brown), micron-scale morphology (males: scales with a rounded apex, females: scales with a dentate apex), and the mechanism of coloration (males: structural color, females: pigment-based color). On the other hand, males and females express almost the same genetic information, with the only difference being the sex of the individual.

The above observations constitute the basis to formulate the following hypothesis: the development of female-type dorsal cover scales is governed by a substance that causes the scale-producing cells of the females to express different genes than the scale-producing cells of the males. Prolonged cooling causes this substance to decompose in certain scales. As the cooling time increases, decomposition takes place in an increasing number of scales, and the changes induced by the decomposition process become increasingly pronounced. In the first step, the photonic nanoarchitecture responsible for the blue color is produced inside the body of the scale, without the alteration of the micron-scale shape of the scale; with longer cooling times, even the micron-scale morphology of the scales is altered. Under a similar duration of cooling, the main effect in males is disordering of the scale rows.

The detailed cellular mechanisms of the production of photonic nanoarchitectures inside the wing scales [18,19] presently are still not well understood. Several papers associate the production of gyroid-type photonic nanoarchitectures with membrane based cellular organelles [35–37], however, as showed by older TEM based [38] and more recent confocal microscopic results [20] the cell morphology of the wing scales is shaped by bundles of microtubules and by F-actin filaments. The changes induced in the brown scales of the female *P*. *icarus* by prolonged cooling may provide a way to achieve deeper insight in the phenomena shaping the interior and the exterior of the scales.

The increase in disorder with increasing cooling time was somewhat surprising because, as shown in recent publications on pupal development monitored under an optical microscope [39], the wing membrane is already present when the pupa is formed, and scale development is initiated shortly after pupation. The scales are produced by specialized cells [21,40] in a well-defined arrangement with socket cells. The examination of uncooled and wild specimens of *P*. *icarus* also showed that the scale-producing cells are arranged in regular rows, making it somewhat intriguing how the disordering observed under cooling times exceeding 6 weeks takes place (S8 Fig). A possible mechanism could be associated with the cellular developmental processes that take place in wing epithelial cells [21] in the pupal stage. Previous work has shown that the arrangement of scale precursor cells closely resembles that of scales in the adult wing [41]. This observation points in the direction of alteration of processes that govern cellular development by prolonged cooling. Further investigations are needed to clarify this mechanism.

The above observations indicate that according to the duration of cooling, the induced changes can be divided into three different periods: less than 4 weeks; 4 to 8 weeks, and more than 8 weeks. We will return to discussing this point in parallel with the eclosion statistics below.

### Structural and spectral changes of the photonic nanoarchitectures

As shown in Fig 5, the deviation of the spectral positions of the blue reflectance maxima is approximately three times wider (35 nm) for females than for noncooled males (10 nm), which is associated with a less-ordered character of the photonic nanoarchitectures, as reported previously [14]. The deviation in the order of 10 nm observed for males is within the range of variation found for a given population [25] and is of a lesser magnitude than the differences found between Europe and Asia [42]. The greater variation of the structural color of the females may arise from the switchover that occurs when scale cells, which would produce brown scales under normal conditions, are forced by prolonged cooling to produce scales with a completely different internal structure: instead of the presence of a mostly empty void within their volume, they develop a sophisticated, layered nanoarchitecture that generates blue color. The dimensions of such nanoarchitecture are very closely linked with the spectral position of the reflectance maximum. Even if simple multilayered model is used [43], the alteration of the layer porosity or layer thickness at the nanometer scale generates a shift in the spectral position of the reflectance maximum. In males, the blue color is used for sexual signaling in each generation [23] and is therefore subjected to selection and presents a narrow range [25]. In females, this selection year by year is not present, and several different genetic variations may be passed with equal probability from generation to generation. Therefore, when cooling switches on the production of the photonic nanoarchitecture, less ordered variants of this nanoarchitecture are produced [14].

The wings and scales of the two males that exhibited the largest spectral difference after 8 weeks of cooling (males #81 and #86) were investigated in more detail. Even with the naked eye, some differences in hue could be perceived between the blue coloration of their dorsal wings, as shown in Fig 7A and 7B. When individual cover scales removed from these wings (Fig 7C, 7D, 7E and 7F) were examined, the scales of male #81 were observed to present a slightly darker shade, and thus, to contain more pigment than the scales of male #86. This may be associated with the modification of the average refractive index of the scale. Comparison of the reflectance spectra of individual whole wings measured either with an integrating sphere or under normal incidence (Fig 8A and 8B) revealed clear differences in the color of the wings. The spectra were normalized to 1 to facilitate comparison. Beyond the shift in the reflectance maximum from 388 nm for male #86 to 416 nm for male #81, the shape of the spectra also differed. These shapes suggested that in male #81, which exhibits a shoulder towards the shorter wavelengths, two slightly different types of nanoarchitectures may be present–side by side–and both of them contribute to the overall reflectance. These differences in the nanoarchitectures of the two males are supported by the averaged reflectance and transmission measurements shown in Fig 8C and 8D, which were performed on the individual scales shown in Fig 7. Here, one must take into account that the measurements presented in Fig 8C and 8D were conducted through a standard optical microscope, which shows reduced transmittance in the UV range that is not perceived by the human eye. Therefore, these spectra cannot be compared to the spectra obtained for whole wings, but they can be compared with each other. Reflectance values over 100% are obtained because the standard against which the measurements were carried out was the microscope slide supporting the scales.

SEM and TEM images of the cover scales of male #81 and #86 are shown in Fig 9. One may clearly observe that the perforations of male #81 exhibit much greater variation in terms of both size and distribution, while the perforations of male #86 are more regular in both their size and distribution. The statistical evaluation of the dimensions of the perforations seen in the SEM images are presented in Fig 9E and 9F. One may note that the linear size of the perforations of male #81 exceed by 20% the size of the perforations of male #86. It has to be taken into account that this linear difference appears squared, when calculating areas. In Fig 9F, the individual measurement data are plotted. One may observe, that a smaller fraction of the holes of male #81 fall in the same range as those of male #86, but the more significant fraction is larger. This can be perceived as two slightly different photonic nanoarchitectures side by side. The unusual shape of the reflectance curve of male #81 seen in Fig 8A, may be attributed to the presence of these two slightly different nanoarchitectures side by side. Such differences in nanoarchitecture may indeed be responsible for the observed differences in optical properties, as we showed previously in the case of two wing surfaces colored blue (dorsal side) and gold-green (ventral side) for *Albulina metallica* butterflies [44].

The measurement of the values of layer thicknesses from the TEM images of males #81 and #86, also indicate small, but well measurable differences (S1 Table). The average thickness of the chitin layers is by 10% larger for male #81 and the air layers separating the chitin layers are by 10% smaller than those of the male #86. These findings very clearly illustrate that differences in dimensions of the order of 10 nm have a clearly observable effect on the optical properties of the photonic nanoarchitecture giving the blue coloration of male *P*. *icarus* butterflies.

### Proportion of eclosion and imperfections as a function of cooling time

Returning to the effects associated with the duration of cooling, the statistics on eclosion and the defective eclosion shown in Fig 10 should be further examined. Here, “cage” indicates the butterflies eclosed from pupae collected from the cage in which the eggs were laid, and the value of “0 weeks” was used for the larvae that were reared in the laboratory after they were collected from the cage but were not subjected to cooling before they were allowed to complete transformation to the adult stage. The fraction of ecdysis was 1 for up to 4 weeks of cooling, at which point a sharp drop to 0.4 was observed, followed by an ecdysis fraction of 0.93 after 6 weeks of cooling. As mentioned above, the pupae that were cooled for 4 weeks were placed in low-light conditions after their removal from the refrigerator to test the effect of light on the resumption of development. The results clearly showed that a sufficiently high level of daylight is needed for the successful resumption of pupal development. Taking into account that we tried to collect fresh pupae before the threshold of ecdysone [28,45] was reached and that, synthesis of ecdysteroids by the prothoracic gland was unlikely during the dark period at 5 °C [46], the doubling of the failure rate of ecdysis observed in the pupae that were allowed to resume development in low-light conditions shows that strong light is needed to properly resume pupal development. For the butterfly *Precis coenia*, measurement of ecdysteroid titers revealed that under long-day conditions, ecdysteroid titers begin to rise 20 h after pupation, while under short-day conditions, ecdysteroid titers do not begin to rise until 60 h after pupation, which is well after the ecdysteroid-critical period is over and leads to seasonal color polyphenism [47]. The increases and decreases in ecdysteroid levels induce sequential bouts of gene expression [48] and thus, affect cellular processes.

Under cooling times ranging from 4 to 8 weeks, the fraction of individuals that eclosed with defects exhibited plateau-like stability at approximately 0.6. After 8 weeks, this fraction started to rise sharply, and at 12 weeks, no defect-free eclosion was observed (Fig 10). The existence of this transitional period corresponded to the disordering of scale arrangement in males (Fig 3A), the increase in the number of the blue scales in females (Fig 3B) and the change in the morphology of the blue scales of females (Fig 4).

## Conclusions

The phenotypic changes induced by the prolonged cooling of freshly formed pupae of *P*. *icarus* butterflies exhibit a high degree of reproducibility. Repeating our previous cooling experiments [14] with more than 200 pupae, we found that, on average, the structural coloration of the dorsal wing surface of the males showed small alterations compared with wild specimens or with butterflies that emerged from pupae that developed to the adult stage in the laboratory and were not subjected to cooling. On the other hand, the average alteration of the pigment-based pattern on the ventral wing surface exhibited changes that were proportional to the duration of cooling. The factor of proportionality was very close to that found in our previous experiments. However, not all individuals that were cooled for a certain duration exhibited a similar magnitude of changes in their ventral wing pattern. This finding may reveal genotypic differences that are normally not apparent under stress-free conditions.

The normally brown females developed an increasing number of blue scales as the cooling time was increased and exhibited greater deviation in the spectral position of the blue reflectance peak associated with blue scales than males. We attribute this difference to the fact that the genes regulating the production of the blue-generating nanoarchitecture of females are not subjected to reproductive selection, in contrast to those of the males [25]. The detailed examination of microscopy data on the arrangement and morphology of scales, together with the statistics on eclosion and the fraction of individuals with defects, support the division of the cooling time into three periods: 0 to 2 weeks, when only moderate changes are induced; 4 to 8 weeks, when significant changes in scale arrangement and morphology are induced, but the fraction of imagines with defects does not exceed 0.6; and 10 to 12 weeks, when the fraction of eclosion decreases to 0 and very few individuals eclose without defects. Detailed spectral and structural analysis of the scales of the males exhibiting the largest difference in structural color after 8 weeks of cooling revealed that the nanoarchitecture of the scales was altered in the specimens showing the greatest deviation from the average value for males. Our data show that deviations in the characteristic dimension of the photonic nanoarchitecture of the order of 10 nm produce easily observable changes in optical properties of the photonic nanoarchitecture responsible for the production of the blue color of male *P*. *icarus* butterflies.

We tentatively attribute the changes observed in terms of the blue scales of the females to the slow decomposition of a substance that governs the development of female-type scales during the cooling. Males and females express the same genetic information with the exception of a “modifier” that accounts for the sex of the individual. In a first step, extending up to 6 weeks of cooling, the external morphology of the brown cover scales of females is not modified, but blue color-generating nanoarchitectures develop in their interior. After 8 weeks of cooling, simultaneous with the increase in the number of blue scales, their external morphology is modified from scales with a dentate apex to scales with a rounded apex (Fig 4), similar in shape to the blue cover scales of males.

In a well-designed cooling experiment conducted with freshly formed pupae of *P*. *icarus* butterflies, we were able to influence complex developmental processes that govern the production of sophisticated nanoarchitectures inside scales possessing structural color and changes in the external shape of the scales.

## Supporting information

S1 Fig Linear fits of all measured quantified aberrations (without averaging) for the two experiments. F-test was carried out on the raw data of the two experiments and showed no significant differences (p = 0.0971) between the two sets.
(TIF)

S2 Fig *Polyommatus icarus* pupae after 10 weeks of cooling at 5 °C in the dark. (TIF)

S3 Fig Ventral wing sides of the *P*. *icarus* butterflies eclosed after 6 weeks of cooling at 5 °C in the dark. First row: males; Rows two, three and four: females.
(TIF)

S4 Fig Blue scales on the dorsal wing surfaces of *P*. *icarus* female specimens. (A) Cooled for 2 weeks in pupal state; (B) cooled for 10 weeks in pupal state.
(TIF)

S5 Fig Ventral wing surfaces of the *P*. *icarus* males cooled for 8 weeks. Specimens (A) #81 and (B) #86 are shown.
(TIF)

S6 Fig *P*. *icarus* pupae which failed to reach eclosion after resuming development following 4 weeks of cooling. The transparent container in which the pupae were allowed to resume development after cooling was placed in low light conditions.
(TIF)

S7 Fig Three *P*. *icarus* butterflies eclosed partially during the cooling (in dark, at 5 °C). The duration of the pupal development was increased from 8–10 days to about 7 weeks. No defect free individual was found.
(TIF)

S8 Fig Optical microscope images of the wings of *P*. *icarus* male specimens where the wing scales of both dorsal and ventral sides were removed. Wild (left) and 10-week-long cooled (right) exemplars are shown in reflected (upper row) and transmitted (lower row) light. On the wild specimen the sockets of the scales are in regular rows while on the cooled specimen disordered arrangement can be seen.
(TIF)

S1 Table Results of the TEM image analysis on *P*. *icarus* males #81 and #86. The thickness of the chitin and air layers were measured on three images on both specimens. The second part of the table shows the average and the standard deviation of the layer thicknesses.
(DOCX)
