# Supplementary figures and images for: Reproducible phenotype alteration due to prolonged cooling of the pupae of Polyommatus icarus butterflies
Source: PLoS One. 2019 Nov 25;14(11):e0225388. doi: 10.1371/journal.pone.0225388 (PMC6876796; doi:10.1371/journal.pone.0225388)

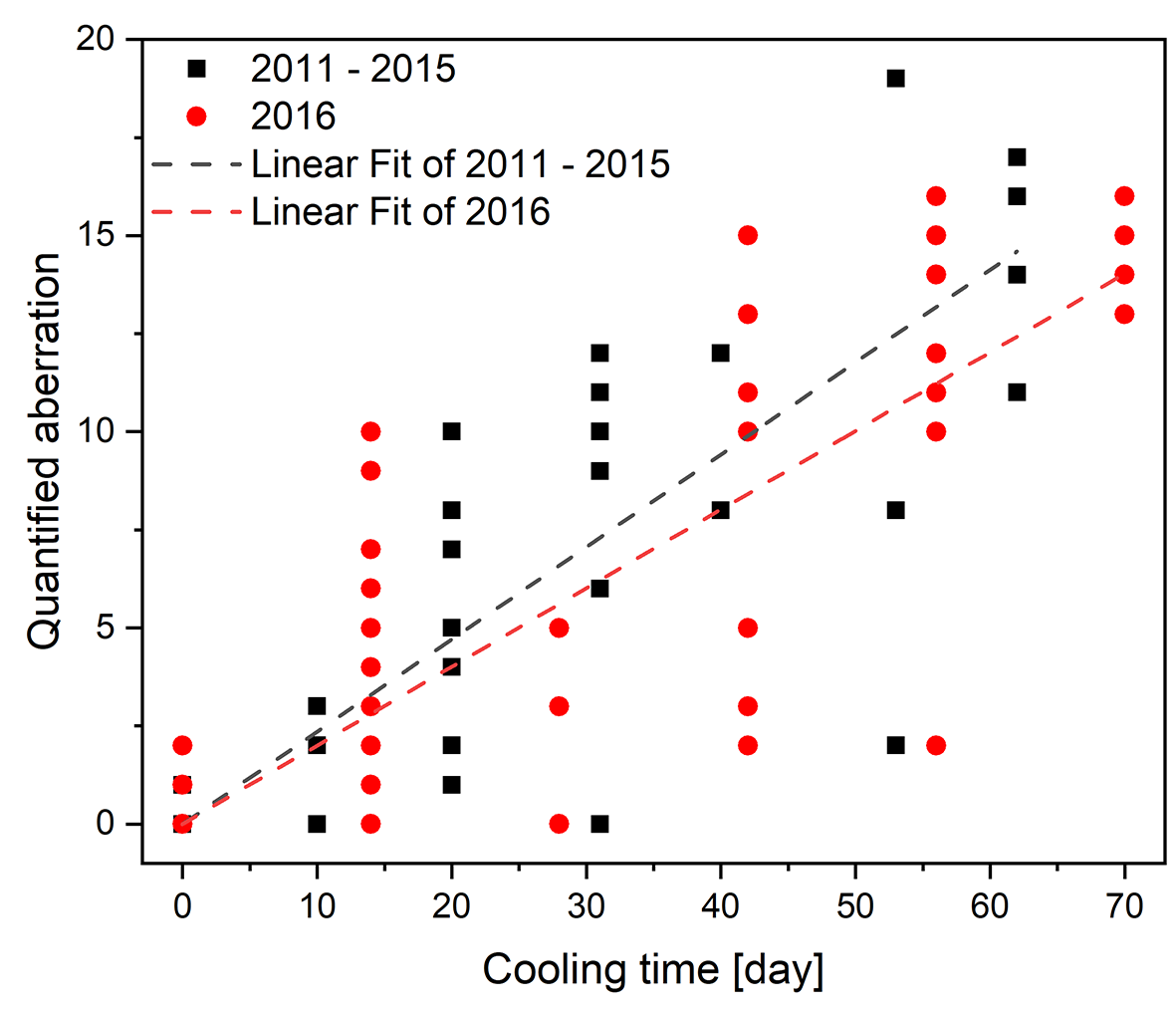

Supplement: S1 Fig — F-test was carried out on the raw data of the two experiments and showed no significant differences (p = 0.0971) between the two sets. (TIF) [file pone.0225388.s001.tif]

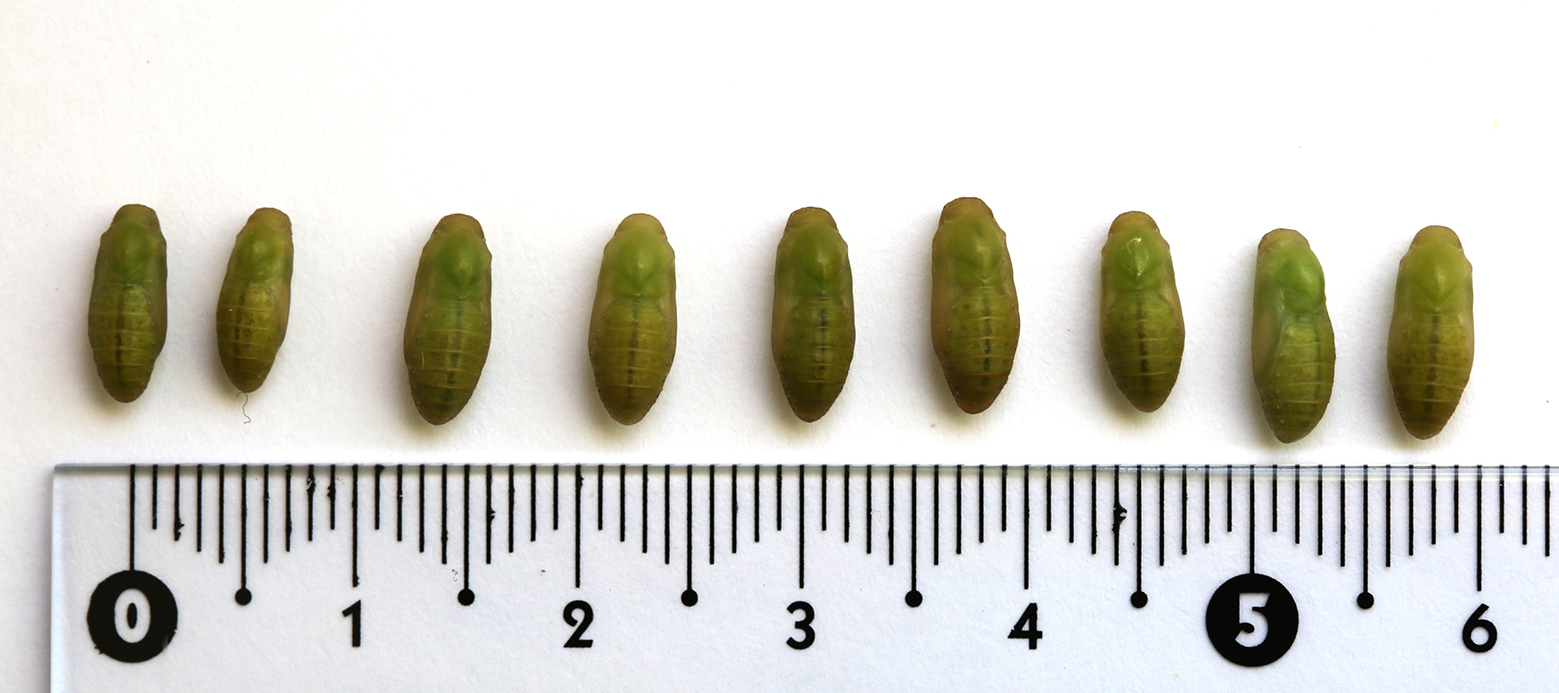

Supplement: S2 Fig — (TIF) [file pone.0225388.s002.tif]

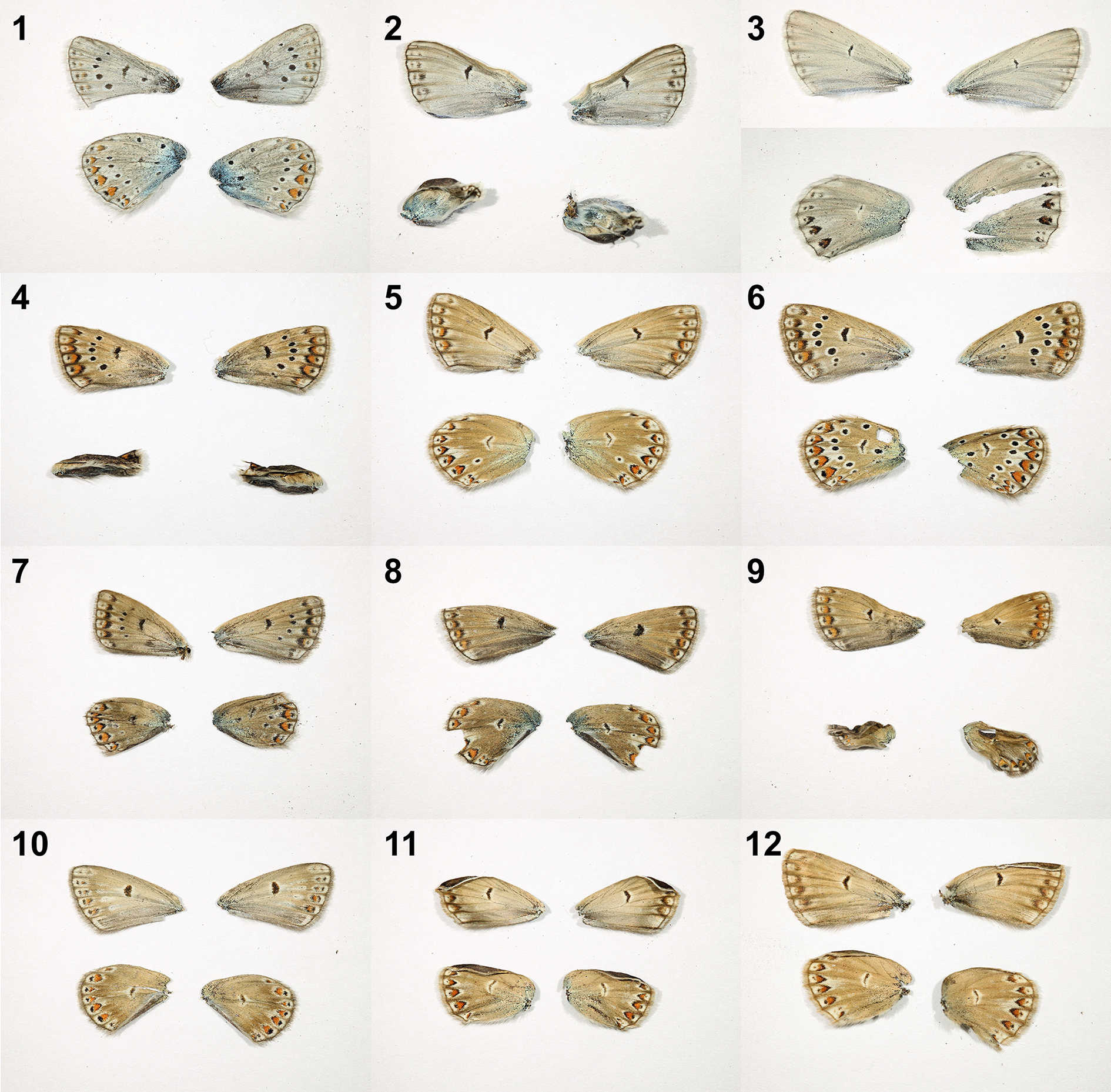

Supplement: S3 Fig — First row: males; Rows two, three and four: females. (TIF) [file pone.0225388.s003.tif]

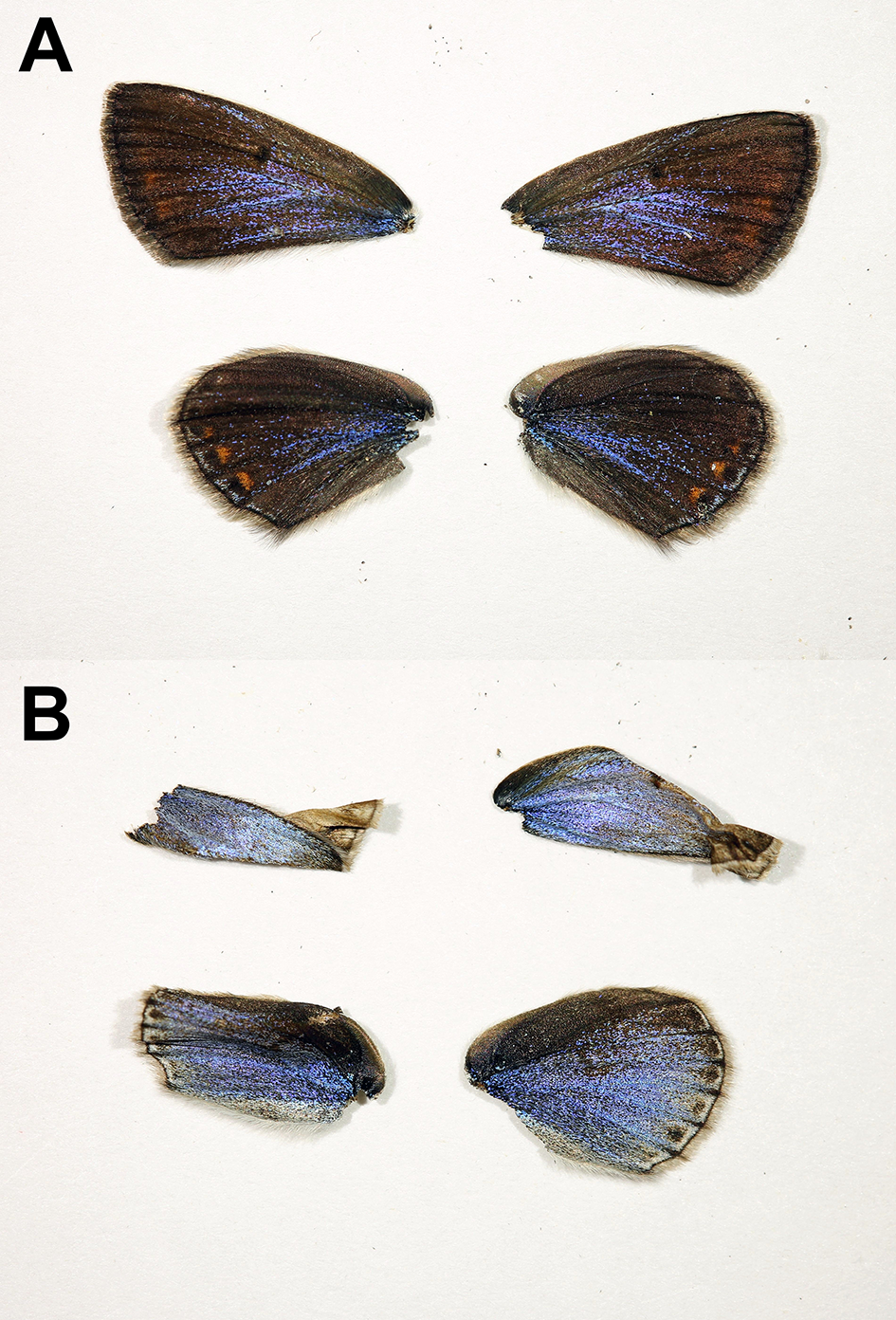

Supplement: S4 Fig — (A) Cooled for 2 weeks in pupal state; (B) cooled for 10 weeks in pupal state. (TIF) [file pone.0225388.s004.tif]

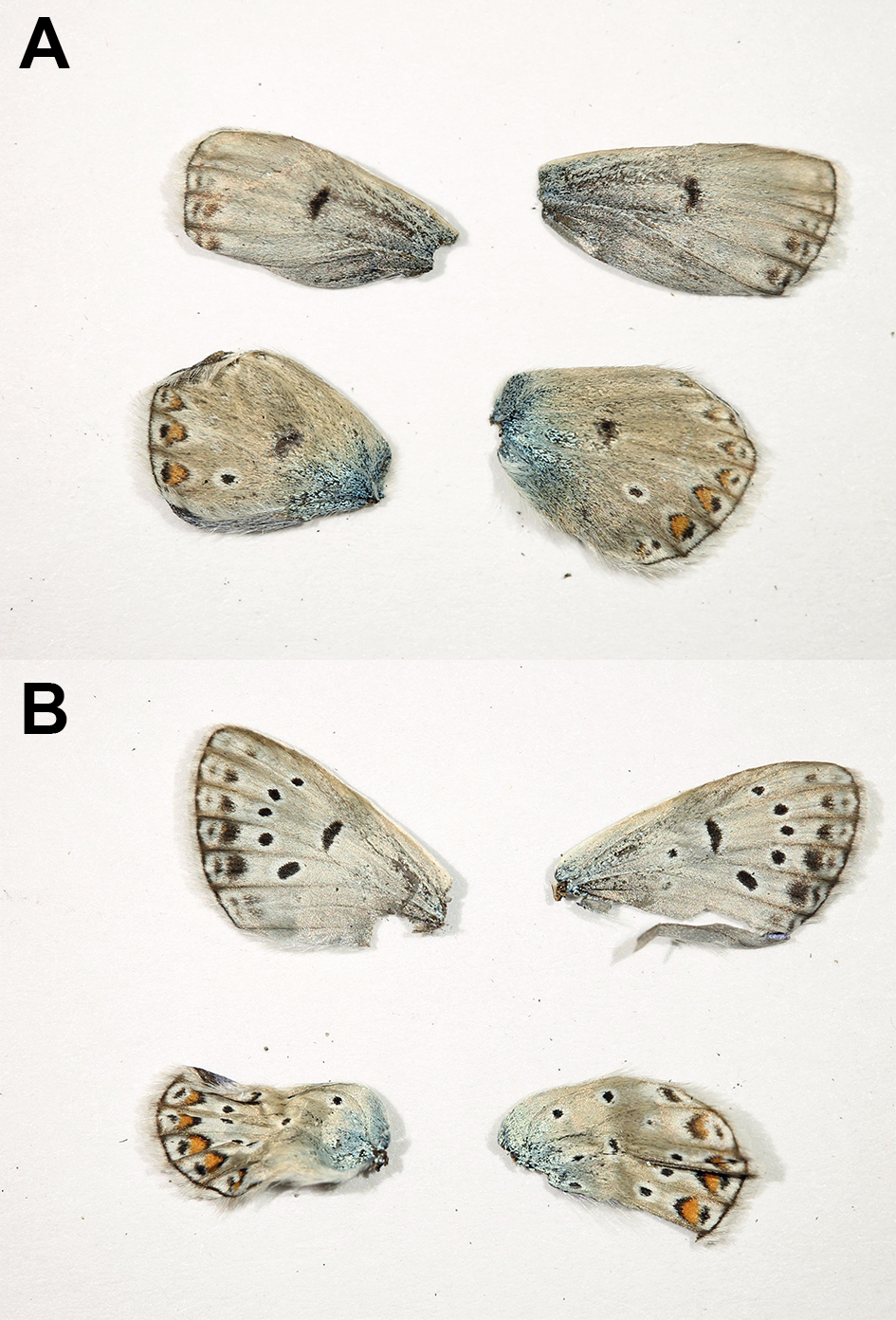

Supplement: S5 Fig — Specimens (A) #81 and (B) #86 are shown. (TIF) [file pone.0225388.s005.tif]

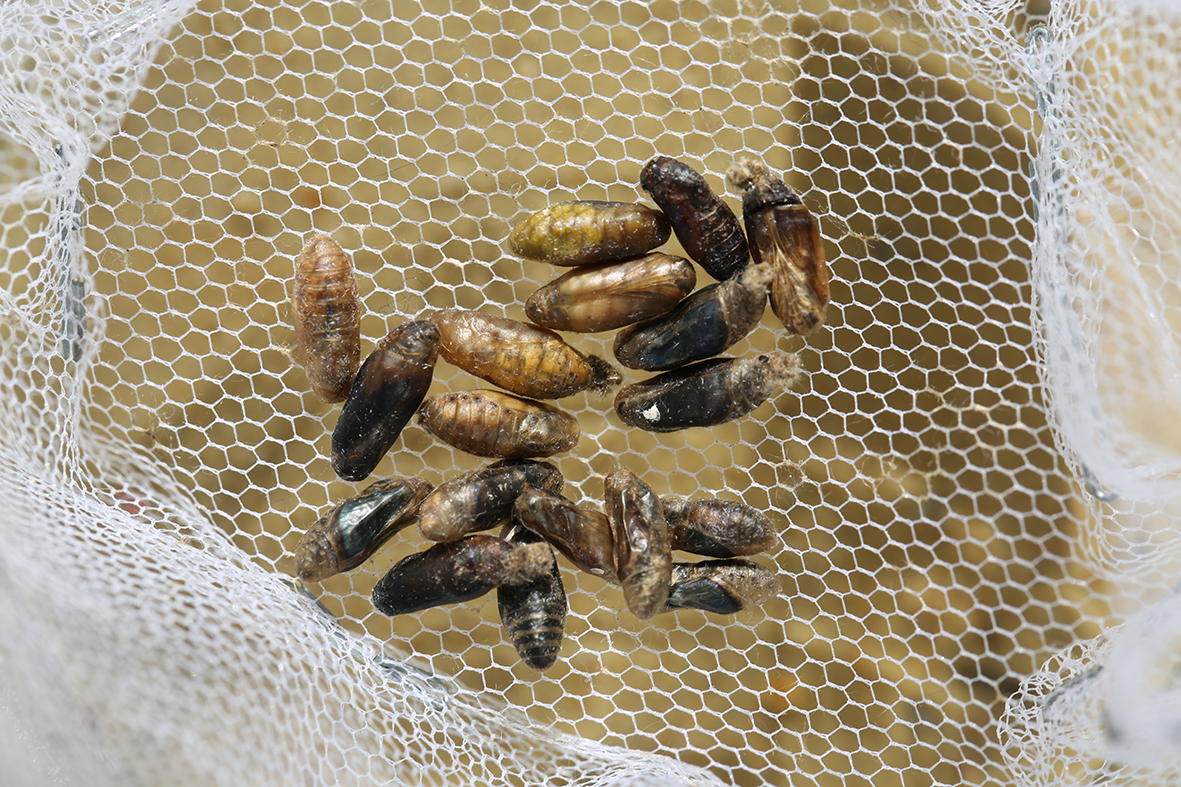

Supplement: S6 Fig — The transparent container in which the pupae were allowed to resume development after cooling was placed in low light conditions. (TIF) [file pone.0225388.s006.tif]

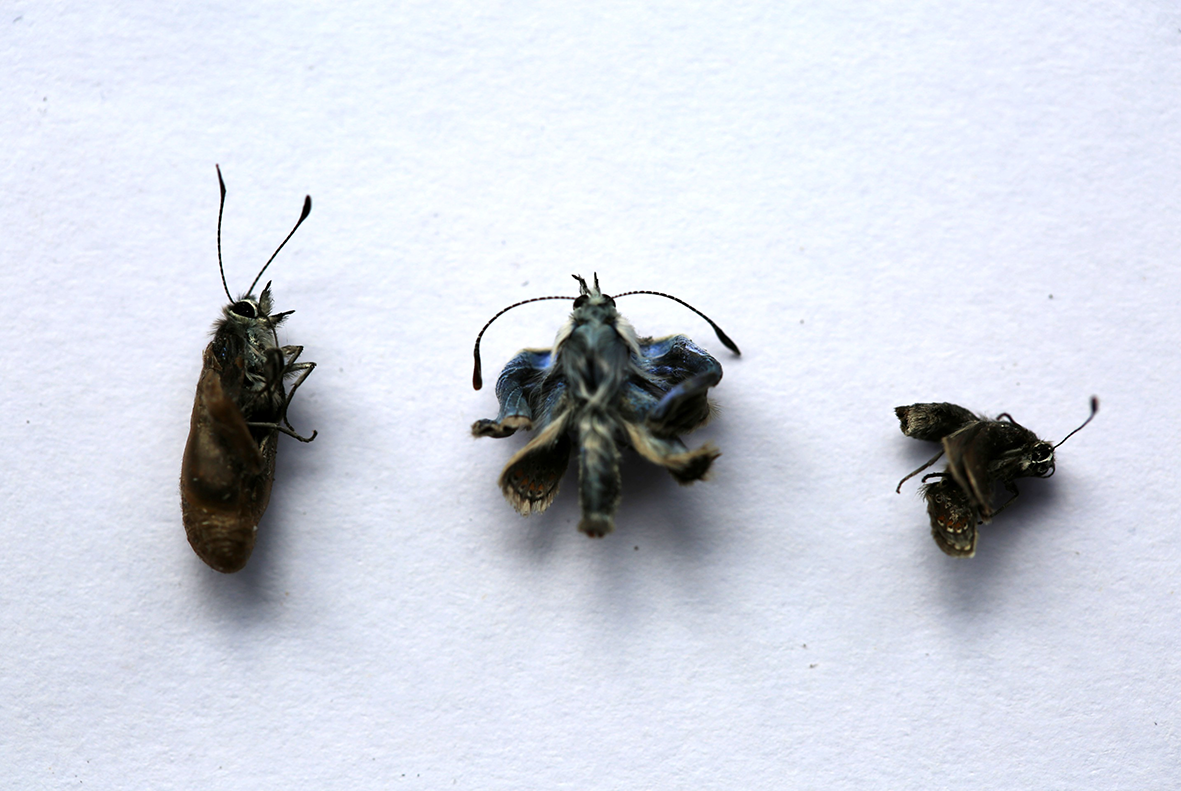

Supplement: S7 Fig — The duration of the pupal development was increased from 8–10 days to about 7 weeks. No defect free individual was found. (TIF) [file pone.0225388.s007.tif]

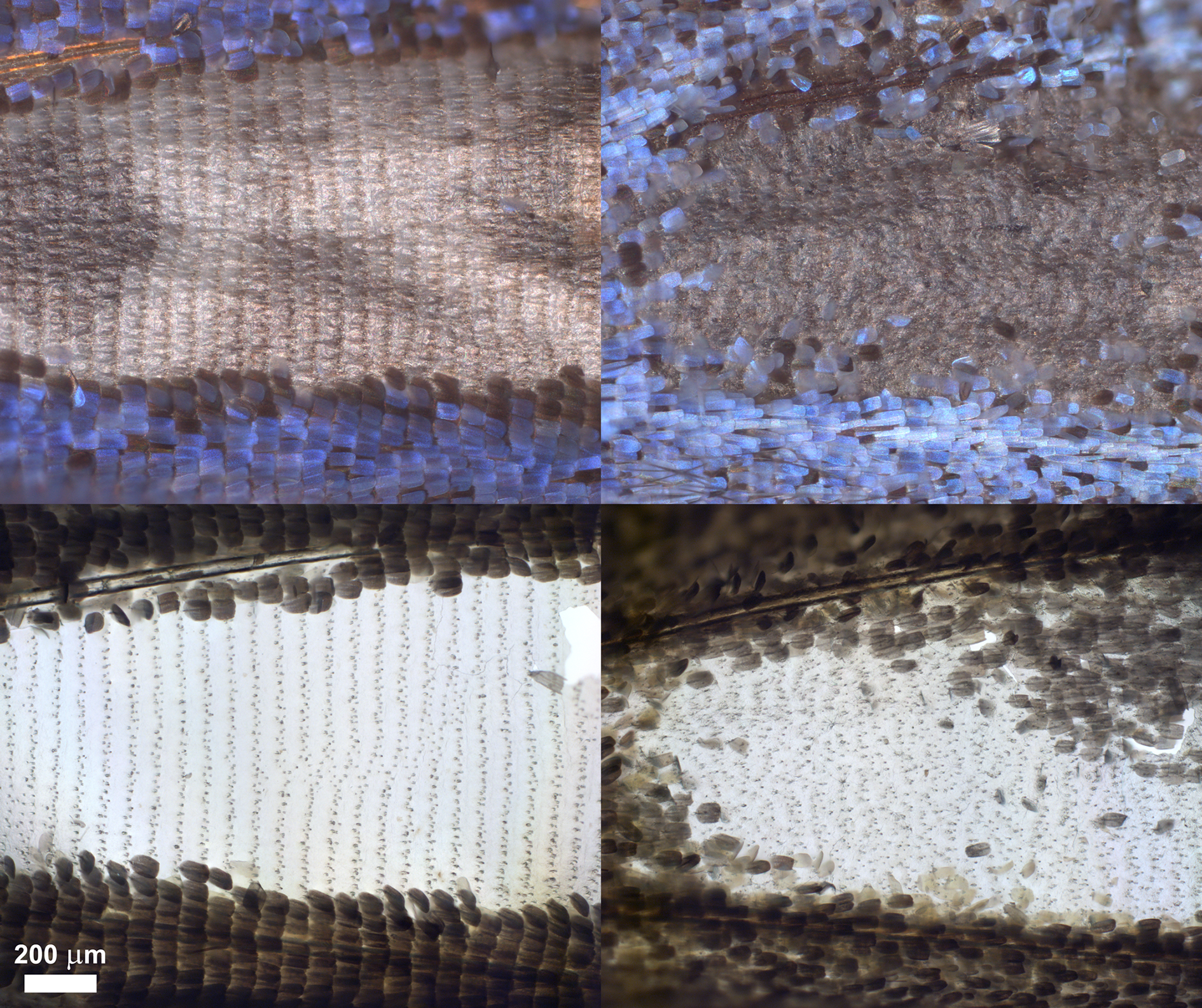

Supplement: S8 Fig — Wild (left) and 10-week-long cooled (right) exemplars are shown in reflected (upper row) and transmitted (lower row) light. On the wild specimen the sockets of the scales are in regular rows while on the cooled specimen disordered arrangement can be seen. (TIF) [file pone.0225388.s008.tif]
