# Supplementary material for: Reproducible phenotype alteration due to prolonged cooling of the pupae of Polyommatus icarus butterflies
Source: PLoS One. 2019 Nov 25;14(11):e0225388. doi: 10.1371/journal.pone.0225388 (PMC6876796; doi:10.1371/journal.pone.0225388)
Supplement: S1 Table — The thickness of the chitin and air layers were measured on three images on both specimens. The second part of the table shows the average and the standard deviation of the layer thicknesses. (DOCX) [file pone.0225388.s009.docx]

|  | **#81 [nm]** | **#86 [nm]** |
| --- | --- | --- |
| Chitin #1 | 73.40 | 64.74 |
| Air #1 | 131.43 | 133.37 |
| Chitin #2 | 80.61 | 72.82 |
| Air #2 | 136.69 | 141.36 |
| Chitin #3 | 74.67 | 68.73 |
| Air #3 | 114.00 | 148.37 |
| Chitin #4 | 68.63 | 64.45 |
|  |  |  |
| avg. Chitin | 74.33 | 67.69 |
| avg. Air | 127.37 | 141.03 |
| dev. Chitin | 4.93 | 3.94 |
| dev. Air | 11.87 | 7.50 |

**S1 Table: Results of the TEM image analysis on *P. icarus* males #81 and #86.** The thickness of the chitin and air layers were measured on three images on both specimens. The second part of the table shows the average and the standard deviation of the layer thicknesses.
